# Isolation and Characterization of Glycosylated Fatty Acid Amides from the Norwegian Deep-Sea Sponge *Phakellia* sp.

**DOI:** 10.3390/md24080264

**Published:** 2026-07-30

**Authors:** Le Ba Vinh, Sindre Wesley Petersen, Diego Rodríguez-Hernández, Pedro A. Ribeiro, Monica Jordheim

**Affiliations:** 1 Department of Chemistry, Faculty of Science and Technology, University of Bergen (UiB), 5007 Bergen, Norway; 2 The Centre for Deep-Sea Research, Department of Biological Sciences (BIO), University of Bergen (UiB), 5006 Bergen, Norway

**Keywords:** Norwegian deep-sea, *Phakellia* sp., glycosylated fatty acid amides, antibacterial screening, biological activity

## Abstract

Marine organisms from deep-sea environments have attracted considerable attention in drug discovery because they produce structurally diverse natural products. However, Norwegian deep-sea ecosystems remain largely unexplored in terms of natural product chemistry and bioactivity. In this study, a deep-sea sponge belonging to the genus *Phakellia* was selectively collected using a low-impact, remotely operated vehicle approach from the Mohn’s Treasure area in the Norwegian Sea at a depth of 2858 m, representing one of the deepest sponge samples investigated in Norwegian waters to date. Chemical investigation of this specimen resulted in the isolation of three new glycosylated fatty acid amides, phakelliosides A–C (**1**–**3**), together with two known compounds, 11-(*S*)-myxillin B (**4**) and 11-(*S*)-myxillin C (**5**), which were isolated as pure individual compounds from natural sources for the first time, with their absolute configurations unambiguously established. These compounds were isolated using a combination of chromatographic techniques, and the structures of the isolated compounds were elucidated by comprehensive spectroscopic analyses, including 1D and 2D NMR and UHPLC–HRMS data. The absolute configurations were determined by optical rotation analysis following acid hydrolysis. Compounds **1**–**5** were subjected to preliminary antibacterial screening against *Enterococcus faecalis, Staphylococcus aureus, Streptococcus agalactiae, Escherichia coli,* and *Pseudomonas aeruginosa* at 100 µg/mL. Under the screening conditions, compound **3** produced the lowest OD_600_ value against *E. faecalis* (OD_600_ = 0.154), whereas generally higher OD_600_ values were observed against the Gram-negative strains. To the best of our knowledge, this is the first report describing the preliminary antibacterial screening of glycosylated fatty acid amides isolated from Norwegian deep-sea organisms. These findings expand current knowledge of the chemical diversity of natural products associated with Norwegian deep-sea sponges. The biosynthetic origin of these metabolites remains unresolved and warrants further investigation.

## 1. Introduction

Marine sponges are among the most prolific and chemically diverse sources of marine natural products [1,2,3,4,5]. While shallow-water sponge biodiversity has been relatively well documented, Arctic deep-sea ecosystems remain poorly explored. These remote and underexplored environments, particularly those in the Norwegian Sea and along the Arctic Mid-Ocean Ridge, host unique sponge species adapted to extreme conditions. However, their biodiversity and natural product chemistry remain poorly understood. Despite increasing interest in these environments, knowledge of their biological composition, ecological roles, and chemical diversity remains limited.

Mohn’s Treasure is a deep-sea feature located along the Arctic Mid-Ocean Ridge, characterized as an inactive sulfide mound at approximately 2600 m depth [6]. Although discovered in 2002, the site remained largely unexplored for more than a decade, and its chemical ecology has not yet been investigated. The first targeted investigation in 2015 used remotely operated vehicles for imaging and sampling [6]. The seafloor is predominantly covered by fine sediments with occasional rocky outcrops, and initial surveys identified 46 morphospecies typical of Arctic bathyal environments, with sponges and echinoderms dominating the assemblage. However, the secondary metabolites produced by organisms in this area have received little chemical investigation.

Mohn’s Treasure and the broader AMOR region remain poorly explored with respect to biodiversity and natural product chemistry. In particular, deep-sea sponges are known to produce structurally diverse and biologically active secondary metabolites, but their diversity and function in this region remain poorly understood [7]. Further multidisciplinary studies integrating biology, ecology, and chemistry are needed to address these knowledge gaps.

**Figure 1:**
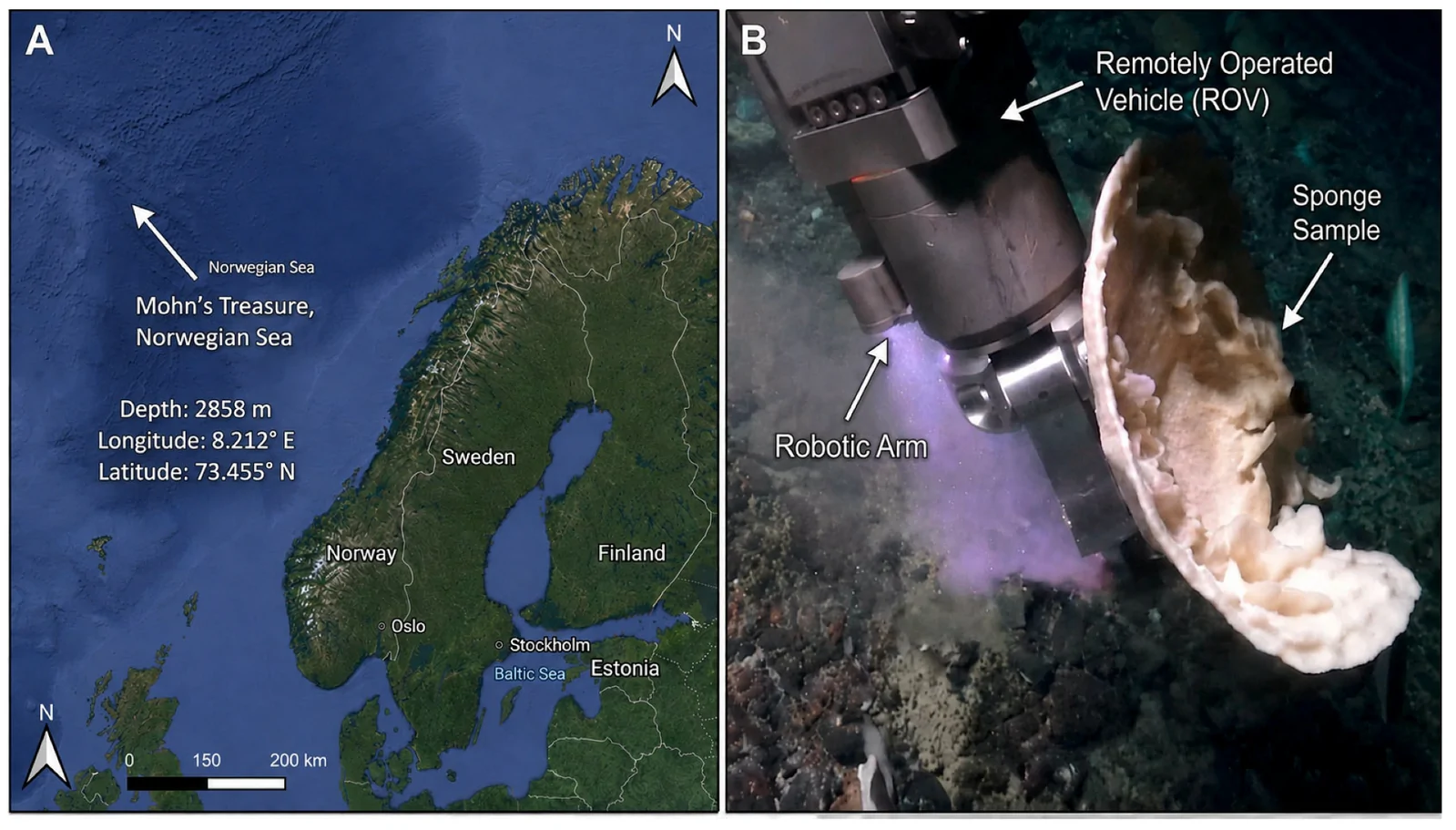
(**A**) Geographical origin of the deep-sea sponge (*Phakellia* sp.) collected from the Mohn’s Treasure area along the Arctic Mid-Ocean Ridge (AMOR) in the Norwegian Sea. The Mohn’s Treasure area is located near 73°44′ N, 07°27′ E, at water depths of approximately 2400–2900 m. The sponge specimen investigated in this study was collected at 73.455° N, 8.212° E and a water depth of 2858 m. (**B**) In situ image of the sponge habitat captured during sampling. A sponge belonging to the genus *Phakellia* was collected using a remotely operated vehicle (ROV, Ægir 6000, Kystdesign AS, Haugesund, Norway), enabling high-resolution imaging and precise sampling at extreme depths. Image credit: UiB/NORMAR.

To date, the biological and ecological characteristics of the Mohn’s Treasure area have been described, whereas information on secondary metabolites produced by organisms inhabiting this region remains limited [6]. In the present study, a deep-sea sponge belonging to the genus *Phakellia* was collected from the Mohn’s Treasure area at a depth of 2858 m (Figure 1). Chemical investigation of this specimen, using a combination of chromatographic techniques, resulted in the isolation of three new glycosylated fatty acid amides (**1**–**3**) along with two known analogues (**4** and **5**). Their structures were subsequently elucidated by comprehensive spectroscopic analyses, including NMR spectroscopy and UHPLC–HRMS.

## 2. Results and Discussion

**Figure 2:**
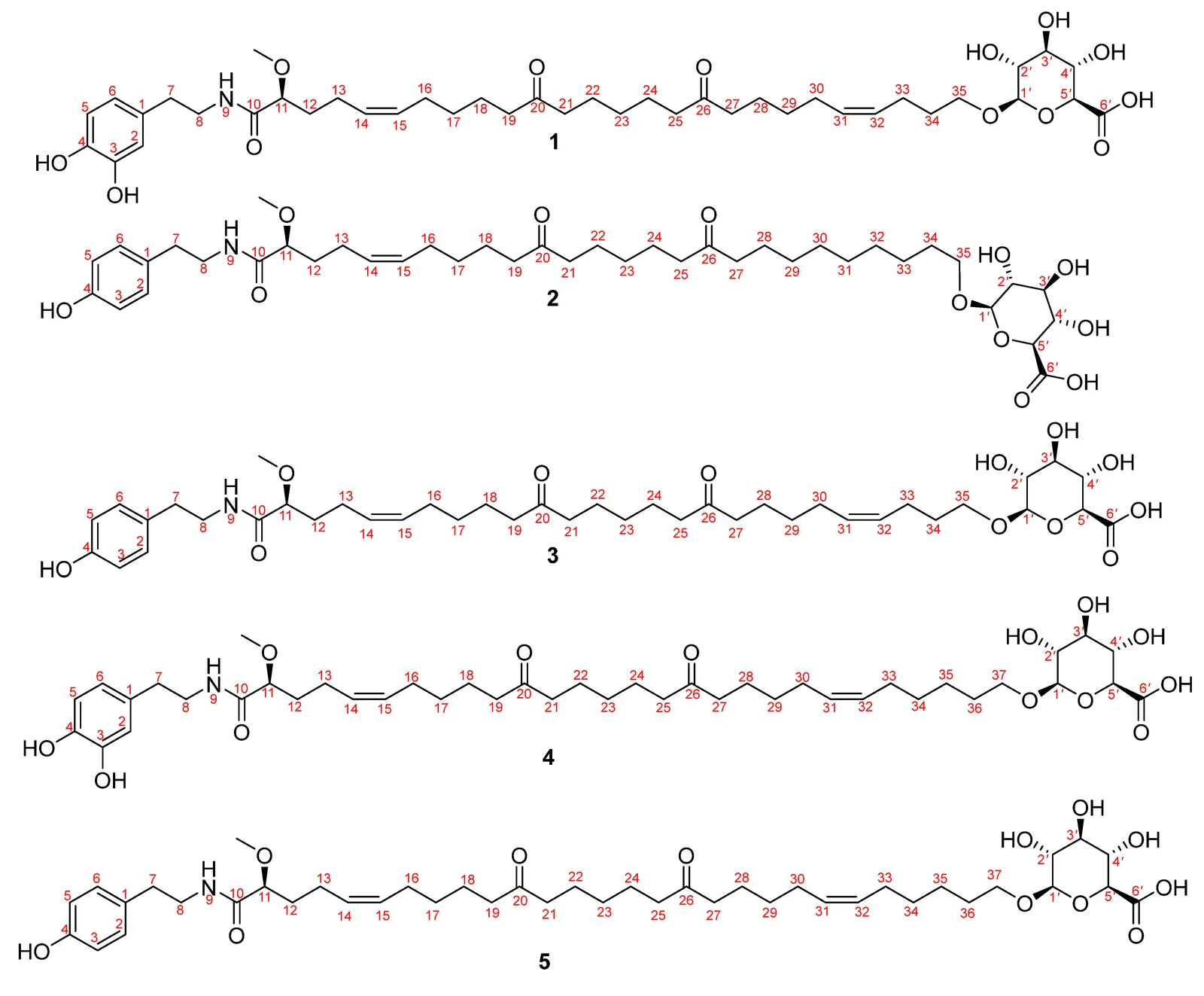
Structures of compounds isolated from the MeOH extract of *Phakellia* sp. The isolated metabolites represent rare gl ycosylated fatty acid amides. The structures were generated using ChemDraw Professional (PerkinElmer, version 22.2.0.3).

Phytochemical investigation of the MeOH extract of *Phakellia* sp. resulted in the isolation of three new compounds, phakelliosides A–C (**1**–**3**), together with two known compounds, 11-(*S*)-myxillin B (**4**) and 11-(*S*)-myxillin C (**5**) (Figure 2). The isolation was achieved using a combination of chromatographic techniques, including silica gel column chromatography, reversed-phase C18 chromatography, and preparative HPLC. The structures of the isolated compounds were elucidated using comprehensive spectroscopic analyses, including NMR and UHPLC–HRMS.

**Figure 3:**
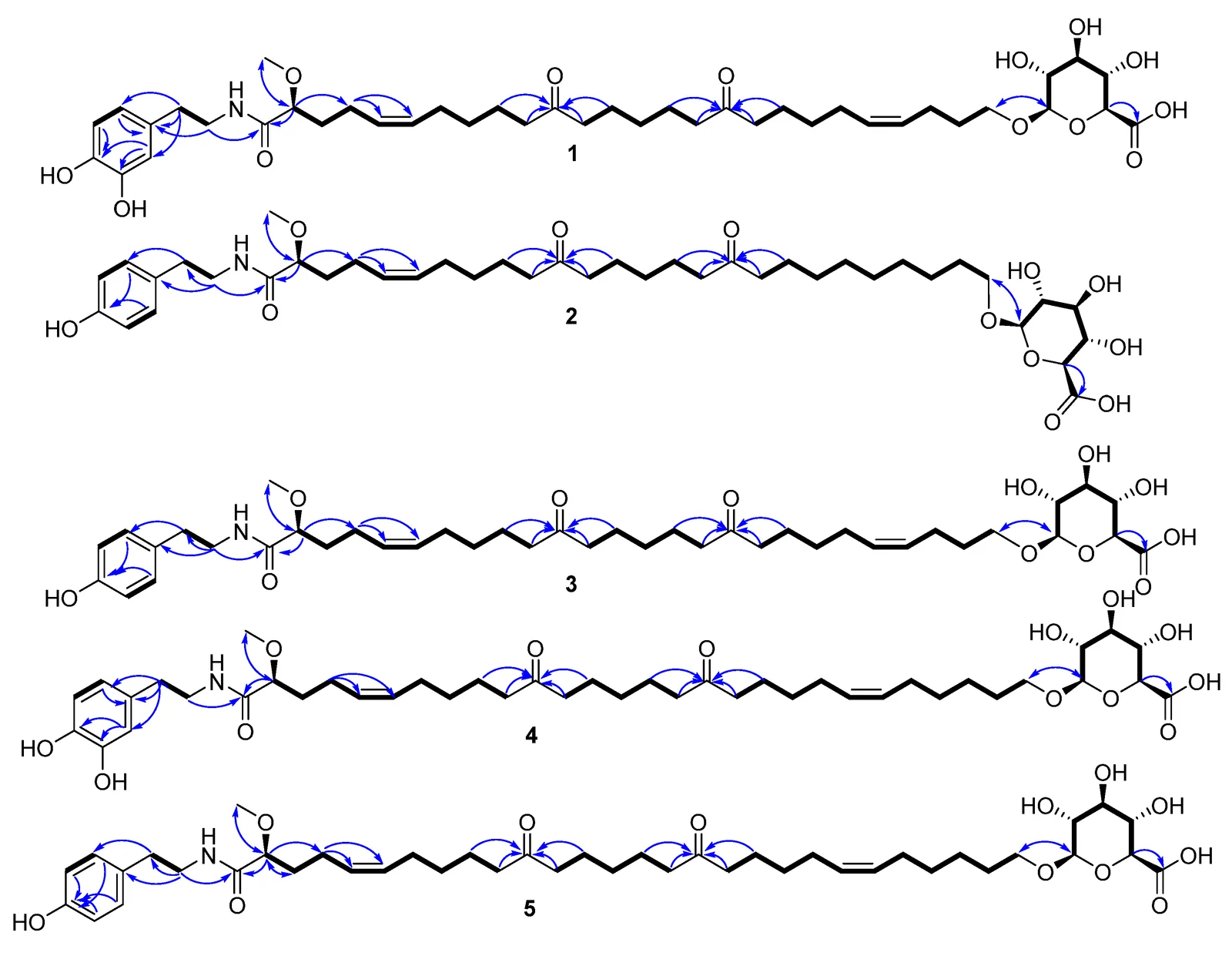
Key ^1^H–^13^C HMBC and ^1^H–^1^H COSY correlations of compounds **1**–**5**. Blue arrows indicate the key HMBC correlations, whereas the bold black lines indicate the ^1^H–^1^H COSY correlations.

**Figure 4:**
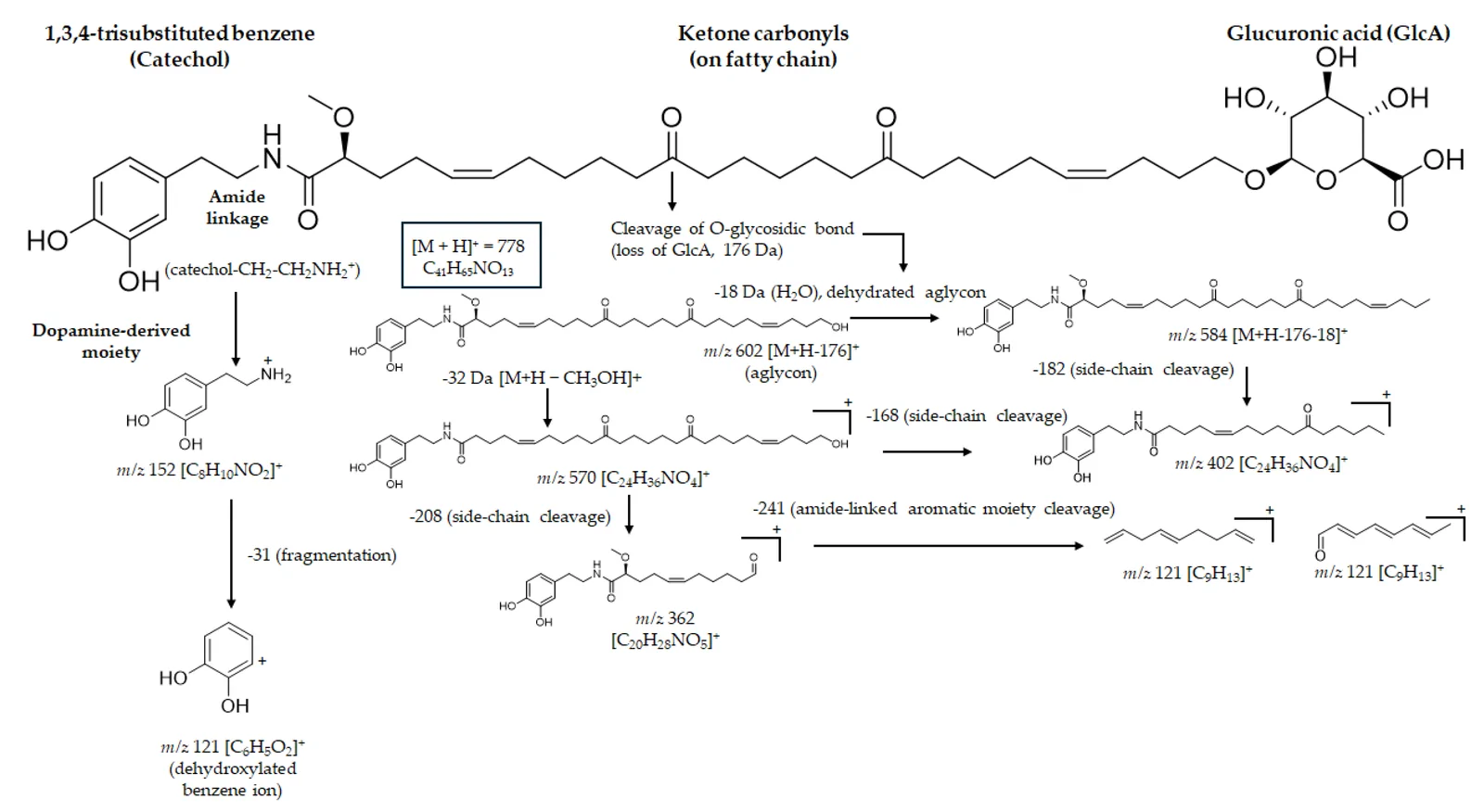
HR-ESI-MS and HR-ESI-MS/MS data of compound **1**, showing characteristic fragmentation pathways derived from detailed analysis of fragment ions and their corresponding neutral losses.

Compound **1** was isolated as a colorless oil. Its optical rotation was measured as [α]_D_^20^-7.5 (*c* 0.05, MeOH). The molecular formula of **1** was established as C_41_H_63_NO_13_ based on HR-ESI-MS data, which showed a protonated ion at *m*/*z* 778.4372 [M + H]^+^, (calcd. 778.4372), and sodiated ion at *m*/*z* 800.4181 [M + Na]^+^, calcd. 800.4192. Compound **1** showed a positive response to Dragendorff’s reagent, indicating the presence of a nitrogen atom. The ^1^H-NMR spectrum (600 MHz, CD_3_OD) of **1** displayed characteristic signals of a 1,3,4-trisubstituted benzene ring, including resonances at *δ*_H_ 6.68 (H, d, *J* = 2.0 Hz, H-2), 6.70 (H, d, *J* = 8.0 Hz, H-5), 6.55 (H, dd, *J* = 8.0, 2.0 Hz, H-6). Additional signals corresponding to a methoxy group at *δ*_H_ 3.30 (3H, s), and an anomeric proton at *δ*_H_ 4.30 (H, d, *J* = 8.0 Hz, H-1′). The ^13^C NMR, and ^1^H–^13^C HSQC spectra revealed 41 carbon signals including, six aromatic carbons at *δ*_C_ 114.9 (C-2), 115.5 (C-5), 119.8 (C-6), 143.5 (C-4), 144.9 (C-3), and 130.4 (C-1), three ketone carbonyl carbons at *δ*_C_ 176.4 (C-10), 211.7 (C-20), and 212.4 (C-26), one carboxylic carbon *δ*_C_ 173.8 (C-6′), and one anomeric carbon at *δ*_C_ 102.9 (C-1′). Two double bonds were identified at *δ*_C_ 127.7 (C-15), 128.5 (C-14), 129.9 (C-31), and 130.5 (C-32). A methoxy carbon signal was observed at *δ*_C_ 57.1 ppm. Analysis of the ^1^H-^1^H COSY and ^1^H-^13^C HSQC spectra indicated the presence of extended aliphatic chains composed predominantly of methylene units. The NMR data of **1** closely resembled those of myxillin A, a previously reported glycosylated fatty acid amide isolated from the sponge *Myxilla incrustans* [8], except for the presence of an additional double bond between C-31 and C-32. Further structural elucidation was achieved through detailed analysis of ^1^H–^13^C HMBC and ^1^H–^1^H COSY correlations. The C7–C8 spin system was assigned to the dopamine-derived moiety. The position of the double bond was established at C-14/C-15 based on COSY correlations and supported by key HMBC cross-peaks. The double bond was assigned a *Z*-configuration, as indicated by the relatively low chemical shift values of the adjacent allylic carbons (*δ*_C_ < 29 ppm) [9,10,11]. Acid hydrolysis confirmed the presence of a glucuronic acid residue (see Supporting Information). The *β*-configuration of the glycosidic linkage was further supported by the coupling constant of the anomeric proton (*J* = 8.0 Hz). Key HMBC correlations from H-1′ (*δ*_H_ 4.30) to C-35 (*δ*_C_ 69.5) established the attachment of the glucuronic acid unit to the aglycone. Additional HMBC and COSY correlations are summarized in Table 1, and the key interactions are shown in Figure 3. The HR-ESI-MS/MS data further supported the proposed structure. The parent ion at *m*/*z* 778 [M + H]^+^ generated a prominent fragment at *m*/*z* 602, corresponding to a neutral loss of 176 Da, consistent with the cleavage of a glucuronic acid (GlcA) moiety ([M + H − 176]^+^). A subsequent ion at *m*/*z* 584 was assigned to dehydration of the aglycone fragment. Further fragment ions at *m*/*z* 570, 534, and 490 were attributed to sequential rearrangement, dehydration, and decarboxylation processes along the oxidized fatty acid chain bearing ketone functionalities. Diagnostic low-*m*/*z* fragments at *m*/*z* 121, 107, and 152 supported the presence of a 1,3,4-trisubstituted catechol ring and its associated ethylamine unit, consistent with a dopamine-derived aromatic moiety. Additional ions at *m*/*z* 189 and 203 were assigned to fragments retaining the catechol–amide portion, confirming the presence of an amide linkage between the aromatic unit and the aliphatic chain. Fragment ions at *m*/*z* 226 and 262 were attributed to cleavage along the fatty acid backbone, while ions at *m*/*z* 402 and 362 arose from further fragmentation of the aglycone framework. The detailed LC–MS/MS fragmentation pathways of **1** are presented in Figure 4. The absolute configuration of the sugar moiety was determined as *β*-D- glucuronic acid based on optical rotation data following hydrolysis and comparison with literature values [12,13]. The aglycon exhibited an optical rotation of [α]_D_^20^ − 8.9 (*c* 0.1, MeOH), consistent with reported data for melonosin B, supporting an 11*S* configuration. Accordingly, compound **1** was identified as a new glycosylated fatty acid amide, designated phakellioside A.

Compound **2** was also obtained as a colorless oil. Its molecular formula was established as C_41_H_65_NO_12_ based on HR-ESI-MS data, which showed a protonated ion at *m*/*z* 764.4573 [M + H]^+^ (calcd for C_41_H_66_NO_12_^+^, 764.4580) and a sodiated ion at *m*/*z* 786.4392 [M + Na]^+^ (calcd for C_41_H_65_NO_12_Na^+^, 786.4399). The 1D and 2D NMR data of compound **2** closely resembled those of compound **1**, except for differences in the substitution pattern of the aromatic ring and the aliphatic chain. Particularly, the 1,3,4-trisubstituted benzene ring observed in **1** was replaced by a para-disubstituted benzene ring in **2**. In addition, the double bond between C-31 and C-32 present in **1** was absent in **2**. These structural differences were supported by key ^1^H–^13^C HMBC correlations and ^1^H–^1^H COSY cross-peaks (Figure 3 and Table 2). The proposed structure was further supported by MS/MS data. Acid hydrolysis of compound **2** afforded D-glucuronic acid, confirming the identity of the sugar moiety (see the section on acid hydrolysis and sugar analysis). The *β*-configuration of the glycosidic linkage was supported by the large coupling constant of the anomeric proton at 4.30 (d, *J* = 8.0 Hz). The absolute configuration at C-11 of the aglycone was determined by comparison of its optical rotation with reported data [9]. After hydrolysis (see Supporting Information), the aglycone exhibited an optical rotation of [α]_D_^20^ − 4.8 (*c* 0.1, MeOH), which is consistent with that of related compounds and supports the assignment of an 11*S* configuration. Compound **2** was identified as a new glycosylated fatty acid amide, designated phakellioside B.

The molecular formula of compound **3** was established as C_41_H_63_NO_12_ based on HR-ESI-MS data, which showed a protonated ion at *m*/*z* 762.4421 [M + H]^+^ (calcd for C_41_H_64_NO_12_^+^, 762.4423) and a sodiated ion at *m*/*z* 784.4240 [M + Na]^+^ (calcd for C_41_H_63_NO_12_Na^+^, 784.4242) (Figure S21, Supporting Information). These data, in conjunction with the ^13^C NMR data (Table 2), confirmed the molecular formula of **3**. The 1D and 2D NMR spectroscopic data of compound **3** closely resembled those of phakellioside B (**2**), with the key difference being the presence of a *Z* double bond between C-31 and C-32. This structural feature was supported by diagnostic ^1^H–^1^H COSY correlations, key ^1^H–^13^C HMBC cross-peaks, and MS/MS data. Acid hydrolysis of compound **3** afforded D-glucuronic acid, confirming the identity of the sugar moiety (see the section on acid hydrolysis and sugar analysis). The *β*-configuration of the glycosidic linkage was supported by the coupling constant of the anomeric proton. The absolute configuration at C-11 of the aglycone was determined by comparison of its optical rotation with literature values. After hydrolysis (see Supporting Information), the aglycone exhibited an optical rotation of [α]_D_^20^ − 6.2 (*c* 0.1, MeOH), consistent with reported data for related compounds and supporting the assignment of an 11S configuration. The presence of 1,3,4-trisubstituted and para-disubstituted aromatic rings, together with a *Z* double bond at C-31/C-32, represents characteristic structural features of this class of glycosylated fatty acid amides and is consistent with the proposed biosynthetic pathway. Based on these data, compound **3** was identified as a new glycosylated fatty acid amide and named phakellioside C.

Compounds **4** and **5** were obtained as colorless oils. Their molecular formulas were established as C_43_H_67_NO_13_ and C_43_H_67_NO_12_, respectively, based on HR-ESI-MS data. Compound **4** showed a protonated ion at *m*/*z* 806.4668 [M + H]^+^ (calcd for C_43_H_68_NO_13_^+^, 806.4685), while compound **5** exhibited a protonated ion at *m*/*z* 790.4732 [M + H]^+^ (calcd for C_43_H_68_NO_12_^+^, 790.4736) and a sodiated ion at *m*/*z* 812.4551 [M + Na]^+^ (calcd for C_43_H_67_NO_12_Na^+^, 812.4555) (Figures S28 and S35, Supporting Information). These data, in conjunction with the ^13^C NMR data (Table 1), confirmed their molecular formulas. The structures of compounds **4** and **5** are consistent with the proposed biosynthetic pathway. In particular, compound **4** featured a 1,3,4-trisubstituted aromatic ring, whereas compound **5** possessed a para-disubstituted aromatic ring. Both compounds shared a common glycosylated fatty acid amide framework composed of a dopamine-derived moiety, a long-chain fatty acid unit, and a glucuronic acid residue. Key structural assignments were supported by diagnostic ^1^H–^13^C HMBC and ^1^H–^1^H COSY correlations (Figure 3 and Supporting Information). Acid hydrolysis of compounds **4** and **5** afforded D-glucuronic acid, confirming the identity of the sugar moiety (see the section on acid hydrolysis and sugar analysis). The *β*-configuration of the glycosidic linkage was supported by the coupling constant of the anomeric proton. The absolute configuration was assigned as 11S by comparison of the specific optical rotation with literature data. After hydrolysis (see Supporting Information), the aglycone fractions exhibited optical rotation values consistent with those of related compounds, supporting the assignment of an 11*S* configuration. Compounds **4** and **5** were previously reported as an inseparable mixture (ratio 2:1) from *Myxilla incrustans* collected at a hydrothermal vent site in Eyjafjordur, northern Iceland [8]; however, they were not isolated as pure individual compounds, and their absolute configurations were not determined. Analysis of compounds as mixtures can hinder accurate structural elucidation and obscure their true biological activities. The isolation of pure compounds enables definitive structural characterization and reliable biological evaluation. This study represents the first successful isolation of compounds **4** and **5** as pure compounds, along with the complete determination of their absolute configurations. Accordingly, compounds **4** and **5** were identified as 11-(*S*)-myxillin B (**4**) and 11-(*S*)-myxillin C (**5**), respectively. These compounds add to the known diversity of sponge-derived fatty acid amides. While their biological function and biosynthetic origin remain unclear, the occurrence of related metabolites in both *M. incrustans* and *M. kobjakovae* indicates a recurring association of this compound class with marine sponges. The biosynthesis and ecological significance of these metabolites remain to be clarified.

Glycolipids, defined as lipids covalently linked to one or more carbohydrate moieties via glycosidic bonds, have been widely reported in marine sponges [14,15]. They are essential components of cellular membranes and are involved in key biological processes, regardless of whether they are produced by the sponge itself or its associated microbiota, including cell recognition, signal transduction, and intercellular communication [16]. Fatty acid amides represent a rare subclass of glycolipids characterized by amide-linked lipid chains bearing glycosyl units. Several compounds from this class have been reported to exhibit biological activities, including anticancer and anti-inflammatory effects. However, the biological properties of this class remain largely unexplored [8,9]. To date, only seven glycosylated fatty acid amides have been reported from marine sponges, including melonosides A–B, melonosins A–B, and myxillins A–C, indicating that only a limited number of members of this class have been identified [8,9,17]. Although myxillins A–C have been structurally characterized, they were reported as an inseparable mixture, likely due to challenges associated with their isolation and purification [8]. This limitation underscores the importance of obtaining pure compounds for definitive structural elucidation and biological evaluation. Among this class, melonoside A was reported to induce autophagy in cisplatin-resistant NCCIT-R germinal tumor cells, whereas myxillin A and myxillin C exhibited anti-inflammatory activities in dendritic cells [9,17]. Despite these reported activities, the currently available pharmacological data for this class remain limited. Knowledge of the biological activities and biosynthesis of these metabolites remains limited. Marine sponges are known to host dense and metabolically active microbial communities, and many sponge-associated secondary metabolites are believed to originate from symbiotic microorganisms. However, the biosynthetic origin of glycosylated fatty acid amides, including melonosides, melonosins, and myxillins, remains unresolved [8,9,17].

Because only limited quantities of the isolated compounds were available (5.9–12 mg per compound), biological evaluation was restricted to a single concentration exploratory screening assay intended to identify trends in bacterial growth inhibition. Compounds **1–5** were evaluated in a preliminary bacterial growth inhibition screening assay at a concentration of 100 µg/mL, and the results are summarized in Table 3. Overall, the compounds produced lower OD_600_ values against Gram-positive strains than against Gram-negative bacteria under the screening conditions. Among the tested compounds, compound **3** gave the lowest OD_600_ value against *Enterococcus faecalis* (OD_600_ = 0.154). Compounds **1**, **2**, and **4** produced OD_600_ values of 0.196–0.207 against this strain, whereas compound **5** produced an OD_600_ value of 0.237. Against *Staphylococcus aureus*, compound **4** produced the lowest OD_600_ value (0.306), followed by compounds **2** and **3**. A similar trend was observed for *Streptococcus agalactiae*, where compound **3** again produced the lowest OD_600_ value (0.367). In contrast, all compounds produced higher OD_600_ values against Gram-negative bacteria. The lowest OD_600_ values against *Escherichia coli* and *Pseudomonas aeruginosa* were obtained for compounds **4** (0.560) and **5** (0.465), respectively. Because this study was limited to a preliminary single-concentration screening assay (100 µg/mL) without MIC determination, dose–response analysis, biological replicates, or statistical evaluation, the present results provide only a preliminary assessment of bacterial growth inhibition and should be interpreted with appropriate caution. Further studies, including MIC determination and dose–response analyses, are required to confirm these observations and explore the structure–activity relationships of this compound class.

Values are expressed as optical density at 600 nm (OD_600_) after incubation at 37 °C for 18–24 h at a concentration of 100 µg/mL. Lower OD_600_ values indicate greater bacterial growth inhibition under the screening conditions. Because of limited compound availability, the reported OD_600_ values represent single measurements obtained in an exploratory screening assay. No biological replicates or statistical analyses were performed. Gentamicin was used as a positive control, with MIC values of 8, 0.06, 4, 0.13, and 0.25 µg/mL against *E. faecalis, S. aureus, S. agalactiae, E. coli,* and *P. aeruginosa*, respectively. A water sample was used as reference control, and medium without bacteria served as the negative control. The final concentration of DMSO in the assay was 0.25% (*v/v*), which showed no effect on bacterial growth.

## 3. Materials and Methods

### 3.1. Deep-Sea Sample Collection and Identification

#### 3.1.1. Geographical Area of Investigation

The Mohn’s Treasure area is located near 73°44′ N, 07°27′ E, approximately 30 km southwest of the active Loki’s Castle vent field, at water depths of 2400–2900 m along the edge of an inner rift wall (Figure 1). Previous surveys of the area provided bathymetric and seafloor data used to characterize the study region. Based on variations in seafloor depth and slope observed in the low-resolution multibeam data available prior to the expedition, the study area was divided into four sampling locations (Figure 1).

Site 1 represents the deepest part of the Mohn’s Treasure area, with a relatively smooth slope at approximately 2810 m depth. Site 2 is located near the beginning of a plateau characterized by very low seafloor gradient and water depths ranging from 2745 to 2750 m. Site 3 is situated in the central part of the plateau at a depth of approximately 2720 m, whereas Site 4 has the steepest gradient and is located at a depth of 2385 m.

#### 3.1.2. Specimen Collection

Sampling was conducted during a University of Bergen research expedition to the Arctic Mid-Ocean Ridge (AMOR) aboard the research vessel *G.O. Sars* in 2023. Deep-sea sponge specimens were collected using the remotely operated vehicle (ROV) Ægir 6000. The *Phakellia* sp. specimen investigated in this study was collected from the Mohn’s Treasure area in the Norwegian Sea (73.455° N, 8.212° E) at a depth of 2858 m.

Immediately after recovery, sponge samples were rinsed with distilled water to remove residual seawater, salts, and surface contaminants. The samples were then allowed to drain and were gently blotted with filter paper to remove excess moisture. Each specimen was placed in a labelled plastic bag, and sample codes were written in pencil on wax paper to prevent smudging during storage. A detailed inventory linking sample codes to collection information was maintained throughout the study. All samples were stored at −80 °C in an ultra-low temperature freezer (SANYO MDF-U33V) until analysis to minimize degradation and preserve chemical integrity. Each *Phakellia* sp. specimen was assigned a unique voucher number. Small tissue samples were preserved in ethanol for subsequent species identification by DNA barcoding.

### 3.2. Biological Material from Deep-Sea Environment

Deep-sea sponge samples were removed from storage, thawed at room temperature, washed thoroughly with distilled water to remove salts and debris, and cut into small pieces prior to extraction. A voucher specimen (PHAKELLIA SP 2023) has been deposited at the Laboratory of Marine Natural Product Chemistry, Department of Chemistry, Faculty of Science and Technology, University of Bergen, and at the Centre for Deep-Sea Research, Department of Biological Sciences, University of Bergen. Detailed extraction and isolation procedures are described in Section 3.4.

### 3.3. General Experimental Procedures

Optical rotations were measured with an Autopol IV polarimeter (Rudolph Research Analytical, Hackettstown, NJ, USA) at 20 °C using the sodium D line (589 nm). Thin-layer chromatography (TLC) was performed on silica gel plates (TLC ALF foils, silica gel 60 with fluorescent indicator at 254 nm; Supelco, Sigma-Aldrich, St. Louis, MO, USA) and RP-18 F254S plates (Supelco, Sigma-Aldrich). Flash chromatography was performed using a puriFlash^®^ XS-420+ system (Interchim, Montluçon, France). Separations were carried out using silica gel (60 Å, 230–400 mesh, 40–63 μm; Supelco, Sigma-Aldrich) and reversed-phase YMC*GEL ODS-A-HG (12 nm, S-50 μm; YMC Co., Kyoto, Japan). Preparative HPLC was performed using an Agilent 1260 Infinity II system (Agilent Technologies, Santa Clara, CA, USA) equipped with a diode-array detector and a YMC Ascentis^®^ C18 column (25 × 2.12 cm, 5 μm; Supelco, Sigma-Aldrich). All purification steps were monitored by 1H NMR spectroscopy to minimize sample loss and ensure reproducible compound recovery (see Supporting Information). TLC was used routinely to monitor separations. Spots were visualized under UV light (254 and 365 nm) and after spraying with 10% aqueous H_2_SO_4_ followed by heating. All solvents and reagents were of HPLC grade and were purchased from Aldrich Chemical Co. (St. Louis, MO, USA), including methanol, acetonitrile, and formic acid.

### 3.4. Extraction and Isolation

The deep-sea sponge *Phakellia* sp. (300 g, wet weight) was cut into approximately 1 cm × 1 cm pieces and freeze-dried for 24 h, yielding 25 g of dry material. The dried sample was extracted with methanol (MeOH, 3 × 0.5 L) at room temperature under sonication for 2 h. The combined extracts were filtered and concentrated under reduced pressure using a rotary evaporator to afford 20 g of crude MeOH extract. The crude extract was subjected to medium-pressure liquid chromatography (MPLC) on a Biotage^®^ Sfär C18 D-Duo column (300 Å, 30 μm) at 17 bar with a flow rate of 25 mL/min, using a stepwise gradient of MeOH–H_2_O (20:80, 40:60, 60:40, 80:20, and 100:0, *v/v*), to yield five fractions (PH-1–PH-5). Fraction PH-4 (240 mg) was further purified by MPLC on a Biotage^®^ Sfär Silica HC D-High-Capacity Duo column (20 μm), eluted with a gradient of MeOH–CH_2_Cl_2_ (5:95, 40:60, 60:40, 95:5, and 100:0, *v/v*), affording thirteen subfractions (PH-4-1–PH-4-13). Subfraction PH-4-12 was preliminarily analyzed by Dragendorff’s reagent, LC–MS (Figure S42), and ^1^H NMR spectroscopy (Figure S40). The ^1^H NMR spectrum of PH-4-12 suggested the presence of glycolipid-like metabolites. In addition, a positive response to Dragendorff’s reagent indicated the presence of nitrogen-containing compounds and suggested the occurrence of glycosylated fatty acid amides in this fraction. Based on these observations, subfraction PH-4-12 was selected for further purification. PH-4-12 (80 mg) was subsequently purified by preparative HPLC (H_2_O/ACN, 62:38, isocratic, flow rate 10 mL/min, UV detection at 205 nm, 20 min), yielding compound **1** (*t*_R_ 6.2, 7.2 mg), compound **2** (*t*_R_ 7.5, 6.6 mg), compound **3** (*t*_R_ 9.6, 6.3 mg), compound **4** (*t*_R_ 12.3, 5.9 mg), and compound **5** (*t*_R_ 16.3, 12.0 mg).

#### Physicochemical and Spectroscopic Data of the Isolated Compounds

Compound **1**. Colorless oil. [α]_D_^20^ − 7.5 (*c* 0.05, MeOH). Molecular formula C_41_H_63_NO_13_ as determined by HR-ESI MS: *m*/*z* 778.4372 [M + H]^+^ (calcd. 778.4372), and *m*/*z* 800.4182 [M+ Na]^+^ (calcd. 800.4192); ^1^H-NMR (600 MHz, CD_3_OD) and ^13^C-NMR (150 MHz, CD_3_OD) are given in Table 1. Detailed ^1^H NMR, ^13^C NMR, ^1^H-^13^C HSQC, ^1^H-^13^C HMBC, and ^1^H-^1^H COSY correlations, as well as LC–MS/MS fragmentation analysis, are provided in the Supporting Information.

Compound **2**. Colorless oil. [α]_D_^20^ − 8.3 (*c* 0.05, MeOH). Molecular formula C_41_H_65_NO_12_ as determined by HR-ESI-MS: *m*/*z* 764.4573 [M + H]^+^ (calcd. 764.4580), and *m*/*z* 786.4392 [M + Na]^+^ (calcd. 786.4399); ^1^H-NMR (600 MHz, CD_3_OD) and ^13^C-NMR (150 MHz, CD_3_OD) are given in Table 1. Detailed ^1^H NMR, ^13^C NMR, ^1^H-^13^C HSQC, ^1^H-^13^C HMBC, and ^1^H-^1^H COSY correlations, as well as LC–MS/MS fragmentation analysis, are provided in the Supporting Information.

Compound **3**. Colorless oil. [α]_D_^20^ − 9.6 (c 0.05, MeOH). Molecular formula C_41_H_63_NO_12_ as determined by HR-ESI-MS: *m*/*z* 762.4421 [M + H]^+^ (calcd. 762.4423), and *m*/*z* 784.4240 [M + Na]^+^ (calcd. 784.4242); ^1^H-NMR (600 MHz, CD_3_OD) and ^13^C-NMR (150 MHz, CD_3_OD) are given in Table 1. Detailed ^1^H NMR, ^13^C NMR, ^1^H-^13^C HSQC, ^1^H-^13^C HMBC, and ^1^H-^1^H COSY correlations, as well as LC–MS/MS fragmentation analysis, are provided in the Supporting Information.

Compound **4**. Colorless oil. [α]_D_^20^ − 8.8 (*c* 0.05, MeOH). Molecular formula C_43_H_67_NO_13_ as determined by HR-ESI MS: *m*/*z* 806.4668 [M + H]^+^ (calcd. 806.4685); ^1^H-NMR (600 MHz, CD_3_OD) and ^13^C-NMR (150 MHz, CD_3_OD) are given in Table 1. Detailed ^1^H NMR, ^13^C NMR, ^1^H-^13^C HSQC, ^1^H-^13^C HMBC, and ^1^H-^1^H COSY correlations, as well as LC–MS/MS fragmentation analysis, are provided in the Supporting Information.

Compound **5**. Colorless oil. [α]_D_^20^ − 7.5 (*c* 0.05, MeOH). Molecular formula C_43_H_67_NO_12_ as determined by HR-ESI MS: *m*/*z* 790.4732 [M + H]^+^ (calcd. 790.4736), and *m*/*z* 812.4551 [M+ Na]^+^ (calcd. 812.4555); ^1^H-NMR (600 MHz, CD_3_OD) and ^13^C-NMR (150 MHz, CD_3_OD) are given in Table 1. Detailed ^1^H NMR, ^13^C NMR, ^1^H-^13^C HSQC, ^1^H-^13^C HMBC, and ^1^H-^1^H COSY correlations, as well as LC–MS/MS fragmentation analysis, are provided in the Supporting Information.

### 3.5. Structure Elucidation

#### 3.5.1. NMR Spectroscopic Analysis

Nuclear magnetic resonance (NMR) spectra, including ^1^H, ^13^C, and two-dimensional experiments, were recorded at ambient temperature on Bruker Avance III spectrometers operating at 600 and 850 MHz. Samples were dissolved in CD_3_OD (0.7 mL). Chemical shifts (*δ*) are reported in parts per million (ppm). Signal multiplicities are denoted as follows: s (singlet), d (doublet), t (triplet), q (quartet), m (multiplet), br (broad), dd (doublet of doublets), and ddd (doublet of doublets of doublets). Coupling constants (*J*) are given in hertz (Hz). All NMR data were processed and analyzed using TopSpin software (Bruker, version 4.5.0) and MestReNova (version 14.0).

#### 3.5.2. Ultra-High-Performance Liquid Chromatography–High-Resolution Mass Spectrometry (UHPLC–HRMS) Analysis

Metabolites isolated from the Norwegian deep-sea sponge *Phakellia* sp. were analyzed using a previously reported method with minor modifications [18,19,20]. Chromatographic analyses were performed on an Agilent 1290 UHPLC system equipped with a Zorbax Eclipse C18 column (50 × 2.1 mm, 1.8 μm). The mobile phases consisted of water containing 0.1% formic acid (solvent A) and acetonitrile containing 0.1% formic acid (solvent B). Separation was achieved using an isocratic elution system (A/B, 62:38, *v*/*v*) at a flow rate of 1.2 mL/min, with a column temperature of 40 °C and an injection volume of 2.0 μL.

High-resolution mass spectrometric data were acquired using an Agilent 6546 Q-TOF mass spectrometer (Agilent Technologies, Santa Clara, CA, USA) equipped with an orthogonal dual electrospray ionization (ESI) source and a reflectron time-of-flight (TOF) analyzer with a dual microchannel plate (MCP) detector. The instrument was operated in positive ion mode with Auto MS/MS acquisition, and the LC effluent was directly introduced at a flow rate of 1.2 mL/min. The operating parameters were as follows: drying gas temperature, 300 °C; capillary voltage, 2500 V; nozzle voltage, 600 V; drying gas flow, 12.0 L/min; nebulizer gas flow, 10 L/min; fragmentor voltage, 200 V; skimmer voltage, 40 V; collision energy, 30 V; and detector voltage, 2400 V. Data were acquired over an *m*/*z* range of 100–3200 with a spectral acquisition rate of 0.5 s. Mass calibration was performed using internal reference standards of known exact masses to ensure mass accuracy, and the mass resolution was approximately 60,000 at *m*/*z* 2200. Data acquisition and analysis were carried out using Agilent MassHunter Workstation Qualitative Analysis software (version 10.0).

### 3.6. Antibacterial Growth Inhibition Assay

The antibacterial activity of the isolated compounds was evaluated using a preliminary bacterial growth inhibition assay based on a previously reported method with minor modifications [21]. Five bacterial strains were included: the Gram-positive species *Staphylococcus aureus*, *Enterococcus faecalis*, and *Streptococcus agalactiae*, and the Gram-negative species *Escherichia coli* and *Pseudomonas aeruginosa*. All strains were obtained from LGC Standards (Teddington, UK). *Staphylococcus aureus*, *E. coli*, and *P. aeruginosa* were cultured in Mueller-Hinton broth, whereas *E. faecalis* and *S. agalactiae* were cultured in brain heart infusion broth. Colonies from blood agar plates were inoculated into the corresponding culture medium and incubated overnight at 37 °C. Cultures were subsequently diluted and incubated further until they reached the logarithmic growth phase. Before the assay, bacterial suspensions were adjusted to 1.5 × 10^3^–1.5 × 10^4^ CFU per well. The assay was performed in 96-well microtiter plates. Samples were dissolved in ddH_2_O and added to each well (50 μL), followed by 50 μL of bacterial suspension to give a final concentration of 100 μg/mL. Plates were incubated overnight at 37 °C, and bacterial growth was assessed by measuring optical density at 600 nm (OD_600_) using a 1420 Multilabel Counter VICTOR3™ (PerkinElmer, Waltham, MA, USA). Distilled water served as the reference control, and medium without bacteria served as the negative control. Gentamicin (32–0.01 μg/mL) was included as a positive control and was visually assessed for inhibition of bacterial growth. Assay validity was confirmed by the expected activity of the positive control. The final concentration of DMSO was 0.25% (*v/v*), which had no detectable effect on bacterial growth. Because of limited compound availability, MIC determination and dose–response studies could not be performed. Accordingly, the reported OD_600_ values represent single measurements obtained in an exploratory screening assay. Biological replicates and statistical analyses were therefore not included. Reference inhibitory concentrations of gentamicin were 8 μg/mL for *E. faecalis*, 0.06 μg/mL for *S. aureus*, 4 μg/mL for *S. agalactiae*, 0.13 μg/mL for *E. coli*, and 0.25 μg/mL for *P. aeruginosa*. Antibacterial assays were conducted at MARBIO (Tromsø, Norway).

## 4. Conclusions

Chemical investigation of the Norwegian deep-sea sponge *Phakellia* sp. resulted in the isolation of five glycosylated fatty acid amides, including three new compounds, phakelliosides A–C (**1**–**3**), together with the first successful purification of the previously reported mixture components 11-(*S*)-myxillin B (**4**) and 11-(*S*)-myxillin C (**5**). The structures of the isolated compounds were elucidated using comprehensive spectroscopic analyses, including NMR and UHPLC–HRMS. The absolute configurations of both the aglycone and sugar moieties were determined by optical rotation following acid hydrolysis. This study expands current knowledge of the metabolites produced by Norwegian deep-sea sponges. The isolated compounds generally produced lower OD_600_ values against Gram-positive than Gram-negative bacteria in a preliminary screening assay conducted at 100 µg/mL. Compound **3** produced the lowest OD_600_ value against *Enterococcus faecalis*, while comparatively lower OD_600_ values were also observed against *Staphylococcus aureus* and *Streptococcus agalactiae*. In general, lower OD_600_ values were observed for Gram-positive bacteria under the screening conditions. Further biological evaluation, including dose–response studies and MIC determination, is needed to assess the biological properties of this compound class.

## Figures and Tables

**Table 1:** NMR spectroscopic data of the isolated compounds (**1**–**5**). The NMR spectra (^1^H, 600 MHz; ^13^C, 150 MHz) were recorded in CD_3_OD.

Position	*δ*_C_	*δ*_H_	*δ*_C_	*δ*_H_	*δ*_C_	*δ*_H_	*δ*_C_	*δ*_H_	*δ*_C_	*δ*_H_
	**1**		**2**		**3**		**4**		**5**	
**1**	130.4	–	129.6	–	129.6	–	130.5	–	129.6	–
**2**	114.9	6.68 (d, *J* = 2.0 Hz)	129.4	7.06 (d, *J* = 8.5 Hz)	129.4	7.06 (d, *J* = 8.5 Hz)	114.9	6.68 (d, *J* = 2.0 Hz)	129.4	7.06 (d, *J* = 8.5 Hz)
**3**	144.9	–	114.9	6.72 (d, *J* = 8.5 Hz)	114.8	6.70 (d, *J* = 8.5 Hz)	144.9	–	114.9	6.72 (d, *J* = 8.5 Hz)
**4**	143.5	–	155.6	–	155.8	–	143.5	–	155.6	–
**5**	115.5	6.70 (d, *J* = 8.0 Hz)	114.9	6.72 (d, *J* = 8.5 Hz)	114.8	6.70 (d, *J* = 8.5 Hz)	115.5	7.05 (d, *J* = 8.0 Hz)	114.9	6.72 (d, *J* = 8.5 Hz)
**6**	119.8	6.55 (dd, *J* = 8.0, 2.0 Hz)	129.4	7.06 (d, *J* = 8.5 Hz)	129.4	7.06 (d, *J* = 8.5 Hz)	119.7	6.55 (dd, *J* = 8.0, 2.0 Hz)	129.4	7.06 (d, *J* = 8.5 Hz)
**7**	34.5	2.68 (td, *J* = 7.0, 1.5 Hz)	34.3	2.74 (td, *J* = 7.0, 1.5 Hz)	34.3	2.69 (td, *J* = 7.0, 1.5 Hz)	34.5	2.68 (td, *J* = 7.0, 1.5 Hz)	34.3	2.74 (td, *J* = 7.0, 1.5 Hz)
**8**	40.3	3.42 (m)	40.3	3.44 (t, *J* = 7.0 Hz)	40.3	3.42 (m)	40.3	3.43 (m)	40.3	3.44 (t, *J* = 7.0 Hz)
**10**	176.4	–	176.3	–	176.2	–	173.8	–	173.8	–
**11**	81.5	3.57 (dd, *J* = 7.5, 4.5 Hz)	81.5	3.56 (dd, *J* = 7.5, 4.5 Hz)	81.5	3.57 (dd, *J* = 7.5, 4.5 Hz)	81.5	3.57 (dd, *J* = 7.5, 4.5 Hz)	81.5	3.57 (dd, *J* = 7.5, 4.5 Hz)
**12**	32.7	1.66 (m)	32.7	1.64 (m)	32.7	1.66 (m)	32.7	1.66 (m)	32.7	1.65 (m)
**13**	22.3	2.07 (m), 2.14 (m)	22.3	2.06 (m), 2.14 (m)	22.3	2.07 (m), 2.14 (m)	22.3	2.07 (m), 2.14 (m)	22.3	2.06 (m), 2.13 (m)
**14**	128.5	5.38 (m)	129.9	5.38 (m)	128.5	5.38 (m)	128.6	5.38 (m)	128.5	5.36 (m)
**15**	127.7	5.33 (m)	128.5	5.35 (m)	127.8	5.33 (m)	127.8	5.33 (m)	130.0	5.38 (m)
**16**	26.5	2.06 (m)	26.8	2.06 (m)	26.6	2.05 (m)	26.8	2.06 (m)	26.7	2.06 (m)
**17**	41.8	2.50 (m)	41.8	2.49 (m)	41.8	2.49 (m)	41.8	2.49 (m)	41.8	2.45 (m)
**18**	22.9	1.52 (m)	22.9	1.53 (m)	22.9	1.53 (m)	22.9	1.53 (m)	22.9	1.53 (m)
**19**	41.9	2.47 (m)	41.9	2.45 (m)	41.9	2.47 (m)	41.9	2.47 (m)	41.9	2.49 (m)
**20**	211.7	–	211.6	–	211.7	–	212.3	–	212.3	–
**21**	41.9	2.47 (m)	41.9	2.45 (m)	41.9	2.47 (m)	41.9	2.47 (m)	41.9	2.49 (m)
**22**	22.8	1.51 (m)	22.9	1.53 (m)	22.9	1.53 (m)	22.9	1.53 (m)	22.9	1.53 (m)
**23**	29.3	1.32 (m)	29.3	1.32 (m)	29.3	1.33 (m)	29.3	1.33 (m)	29.3	1.32 (m)
**24**	26.7	2.06 (m)	26.7	2.06 (m)	26.7	2.06 (m)	26.7	2.06 (m)	26.7	2.06 (m)
**25**	42.0	2.48 (m)	42.0	2.46 (m)	42.0	2.48 (m)	42.0	2.48 (m)	42.0	2.46 (m)
**26**	212.4	–	212.3	–	212.4	–	211.7	–	211.6	–
**27**	42.0	2.48 (m)	42.0	2.46 (m)	42.0	2.48 (m)	42.0	2.48 (m)	42.0	2.46 (m)
**28**	23.1	1.53 (m)	23.1	1.55 (m)	23.1	1.55 (m)	23.1	1.55 (m)	23.1	1.55 (m)
**29**	28.9	1.32 (m)	28.9	1.32 (m)	28.9	1.32 (m)	28.9	1.32 (m)	28.9	1.34 (m)
**30**	26.5	2.06 (m)	26.5	2.06 (m)	26.5	2.06 (m)	26.5	2.06 (m)	26.5	2.06 (m)
**31**	129.9	5.39 (m)	23.0	1.55 (m)	129.9	5.39 (m)	129.4	5.39 (m)	130.5	5.31 (m)
**32**	130.5	5.41 (m)	25.7	1.40 (m)	130.6	5.41 (m)	130.0	5.41 (m)	127.8	5.39 (m)
**33**	25.6	1.41 (m)	25.6	1.38 (m)	25.6	1.39 (m)	25.7	1.39 (m)	25.7	1.37 (m)
**34**	29.4	1.64 (m)	29.2	1.63 (m)	29.4	1.64 (m)	29.4	1.64 (m)	29.4	1.63 (m)
**35**	69.5	3.53 (m), 4.00 (m)	42.1	2.44 (m)	69.7	3.53 (m), 3.96 (m)	29.3	3.53 (m), 3.96 (m)	29.3	1.33 (m)
**36**	–	–	23.5	1.57 (m)	–	–	23.5	1.59 (m)	29.2	1.33 (m)
**37**	–	–	69.8	3.54 (m), 3.95 (m)	–	–	69.8	3.54 (m), 3.96 (dd, *J* = 17.0, 7.0 Hz)	69.8	3.54 (m), 3.95 (dd, *J* = 16.0, 9.0 Hz)
**1′**	102.9	4.27 (d, *J* = 8.0 Hz)	103.0	4.30 (d, *J* = 8.0 Hz)	103.0	4.30 (d, *J* = 8.0 Hz)	103.0	4.30 (d, *J* = 7.0 Hz)	103.0	4.30 (d, *J* = 8.0 Hz)
**2′**	73.6	3.23 (t, *J* = 8.5 Hz)	73.5	3.24 (t, *J* = 8.5 Hz)	73.5	3.23 (m)	73.5	3.24 (t, *J* = 8.5 Hz)	73.5	3.24 (t, *J* = 8.0 Hz)
**3′**	74.7	3.56 (m)	74.9	3.55 (m)	75.0	3.65 (d, *J* = 9.5 Hz)	76.4	3.43 (m)	76.4	3.43 (m)
**4′**	72.4	3.41 (m)	72.3	3.43 (m)	72.3	3.48 (m)	72.3	3.48 (m)	72.3	3.48 (m)
**5′**	76.5	3.45 (t, *J* = 9.0 Hz)	76.4	3.45 (m)	76.4	3.43 (m)	74.9	3.66 (d, *J* = 9.5 Hz)	74.9	3.66 (d, *J* = 9.0 Hz)
**6′**	173.8	–	173.8	–	173.8	–	176.5	–	176.4	–
**OCH_3_**	57.1	3.30 (s)	57.1	3.30 (s)	57.1	3.30 (s)	57.1	3.30 (s)	57.1	3.30 (s)

Chemical shifts (*δ*) are reported in ppm and coupling constants (*J*) in Hz and are rounded to one decimal place. Assignments were established based on DEPT, ^1^H-^1^H COSY, ^1^H-^13^C HSQC NMR, and ^1^H-^13^C HMBC NMR experiments. Several overlapping signals, particularly those of CH_2_ groups, may be interchangeable.

**Table 2:** Chemical profile of glycosylated fatty acid amides identified in the metabolite-rich fraction PH-4-12 of the sponge *Phakellia* sp.

Compounds	*t*_R_ (mins)	Observed *m*/*z*	Calculated *m*/*z*	Error *m*/*z* (ppm)	Molecular Formular [M + H]^+^	MS/MS Fragments (*m*/*z*)	Collision Energy (eV)	UV (λ_max_, nm)	Compound No
Phakellioside A	1.9	778.4372	778.4372	0	C_41_H_64_NO_13_^+^	602[M + H − C_6_H_8_O_6_]^+^	30	200	**1**
Phakellioside C	2.0	762.4421	762.4423	−0.26	C_41_H_64_NO_12_^+^	586[M + H − C_6_H_8_O_6_]^+^	30	200	**3**
Phakellioside B	2.9	764.4573	764.4580	−0.92	C_41_H_66_NO_12_^+^	588[M + H − C_6_H_8_O_6_]^+^	30	200	**2**
11-(*S*)-myxillin B	3.6	806.4668	806.4685	−2.11	C_43_H_68_NO_13_^+^	630[M + H − C_6_H_8_O_6_]^+^	30	200	**4**
11-(*S*)-myxillin C	4.9	790.4732	790.4736	−0.51	C_43_H_68_NO_12_^+^	614[M + H − C_6_H_8_O_6_]^+^	30	200	**5**

**Table 3:** Preliminary antibacterial screening of compounds **1**–**5** at 100 µg/mL. Tested bacterial strains: *Enterococcus faecalis*, *Staphylococcus aureus*, *Streptococcus agalactiae* (Streptococcus B), *Escherichia coli*, and *Pseudomonas aeruginosa*. Assays were performed in 96-well microtiter plates using Mueller–Hinton broth (*S. aureus*, *E. coli*, *P. aeruginosa*) or brain heart infusion broth (*E. faecalis*, *S. agalactiae*).

Compound	*E. faecalis*	*S. aureus*	*S. agalactiae*	*E. coli*	*P. aeruginosa*
**1**	0.207	0.344	0.375	0.580	0.497
**2**	0.202	0.327	0.416	0.636	0.532
**3**	0.154	0.326	0.367	0.590	0.589
**4**	0.196	0.306	0.456	0.560	0.468
**5**	0.237	0.349	0.478	0.610	0.465

## Data Availability

The data supporting the findings of this study are available within the article and its Supplementary Materials.

## Supplementary Materials

The following supporting information can be downloaded at https://www.mdpi.com/article/10.3390/md24080264/s1: Acid hydrolysis and determination of the absolute configuration of the aglycone and sugar moieties, NMR spectra, HR-ESI-MS and HR-ESI-MS/MS data, as well as 1H NMR spectra of the screening-guided fractions for all isolated compounds, are provided in Figures S1–S42.
