# Supplementary material for: Isolation and Characterization of Glycosylated Fatty Acid Amides from the Norwegian Deep-Sea Sponge Phakellia sp
Source: Mar Drugs. 2026 Jul 30;24(8):264. doi: 10.3390/md24080264 (PMC13514762; doi:10.3390/md24080264)
Supplement: Supplementary file 1 [file marinedrugs-24-00264-s001.zip › marinedrugs-4431401-supplementary.pdf]

Contents

Acid Hydrolysis and Determination of the Absolute Configuration of the Aglycone and Sugar Moieties 4

Figure S1: ^{1}H NMR spectrum (CD_3OD, 600 MHz) of compound **1** 5

Figure S2: ^{13}C NMR spectrum (CD_3OD, 150 MHz) of compound **1** 6

Figure S3: ^{1}H-^{13}C HSQC spectrum (CD_3OD, 600 MHz) of compound **1** 7

Figure S4: ^{1}H-^{1}H COSY spectrum (CD_3OD, 600 MHz) of compound **1** 8

Figure S5: ^{1}H-^{13}C HMBC spectrum (CD_3OD, 600 MHz) of compound **1** 9

Figure S6: HR-ESI-MS spectrum of compound **1** 9

Figure S7: HR-ESI-MS/MS spectrum of compound **1** 10

Figure S8: ^{1}H NMR spectrum (CD_3OD, 600 MHz) of compound **2** 10

Figure S9: ^{13}C NMR spectrum (CD_3OD, 150 MHz) of compound **2** 11

Figure S10: ^{1}H-^{13}C HSQC spectrum (CD_3OD, 600 MHz) of compound **2** 12

Figure S11: ^{1}H-^{13}C HMBC spectrum (CD_3OD, 600 MHz) of compound **2** 13

Figure S12: ^{1}H-^{1}H COSY spectrum (CD_3OD, 600 MHz) of compound **2** 14

Figure S13: HR-ESI-MS spectrum of compound **2** 14

Figure S14: HR-ESI-MS/MS spectrum of compound **2** 15

Figure S15: ^{1}H NMR spectrum (CD_3OD, 600 MHz) of compound **3** 15

Figure S16: ^{13}C NMR spectrum (CD_3OD, 150 MHz) of compound **3** 16

Figure S17: DEPT spectrum (CD_3OD, 150 MHz) of compound **3** 17

Figure S18: ^{1}H-^{13}C HSQC spectrum (CD_3OD, 600 MHz) of compound **3** 18

Figure S19: ^{1}H-^{13}C HMBC spectrum (CD_3OD, 600 MHz) of compound **3** 19

Figure S20: ^{1}H-^{1}H COSY spectrum (CD_3OD, 600 MHz) of compound **3** 20

Figure S21: HR-ESI-MS spectrum of compound **3** 20

Figure S22: HR-ESI-MS/MS spectrum of compound **3** 21

Figure S23: ^{1}H NMR spectrum (CD_3OD, 600 MHz) of compound **4** 21

Figure S24: ^{13}C NMR spectrum (CD_3OD, 150 MHz) of compound **4** 22

Figure S25: ^{1}H-^{13}C HSQC spectrum (CD_3OD, 600 MHz) of compound **4** 23

Figure S26: ^{1}H-^{13}C HMBC spectrum (CD_3OD, 600 MHz) of compound **4** 24

Figure S27: ^{1}H-^{1}H COSY spectrum (CD_3OD, 600 MHz) of compound **4**.......................... 25

Figure S28: HR-ESI-MS spectrum of compound **4** 25

Figure S29: HR-ESI-MS/MS spectrum of compound **4** 26

Figure S30: ^{1}H NMR spectrum (CD_3OD, 600 MHz) of compound **5** 27

Figure S31: ^{13}C NMR spectrum (CD_3OD, 150 MHz) of compound **5**................................ 28

Figure S32: ^{1}H-^{13}C HSQC spectrum (CD_3OD, 600 MHz) of compound **5** 29

Figure S33: ^{1}H-^{1}H COSY spectrum (CD_3OD, 600 MHz) of compound **5**.......................... 30

Figure S34: ^{1}H-^{13}C HMBC spectrum (CD_3OD, 600 MHz) of compound **5** 31

Figure S35: HR-ESI-MS spectrum of compound **5** 31

Figure S36: HR-ESI-MS/MS spectrum of compound **5** 32

Figure S37: ^{1}H NMR spectrum for screening-guided isolation of fraction **PH-2**............ 32

Figure S38: ^{1}H NMR spectrum for screening-guided isolation of fraction **PH-3**............ 33

Figure S39: ^{1}H NMR spectrum for screening-guided isolation of fraction **PH-4**............ 34

Figure S40: ^{1}H NMR spectrum for screening-guided isolation of fraction **PH-5**............ 35

Figure S41: ^{1}H NMR spectrum for screening-guided isolation of fraction **PH-4-12**........ 36

Figure S42: Secondary metabolites present in subfraction **PH-4-12** of the sponge *Phakellia* sp. were profiled using LC–QTOF–MS/MS in positive ionization mode. (A) Total ion chromatogram (TIC) acquired in positive mode. (B) Ultraviolet (UV, 220 nm) chromatogram of subfraction **PH-4-12**. 37

Acid Hydrolysis and Determination of the Absolute Configuration of the Aglycone and Sugar Moieties

Each compound (2.0 mg) was separately dissolved in 2.0 M HCl (dioxane/H_2O, 1:1, v/v, 1.0 mL) and heated at 80 °C in a water bath for 6 h. After cooling to room temperature, the reaction mixtures were partitioned between CH_2Cl_2 and H_2O to separate the aglycone and sugar fractions. The organic (CH_2Cl_2) layer containing the aglycone and the aqueous layer containing the sugar moiety were collected and evaporated to dryness under a stream of N_2. The isolated sugar residues were dissolved in H_2O and allowed to stand for 24 h prior to measurement of optical rotation values, which were compared with literature data. The sugars obtained from compounds **1–5** were identified as D-glucuronic acid (1.0 mg), $[\alpha]_D^{20}$ +9.8 (c 0.1, H_2O), consistent with the reported value ($[\alpha]_D^{25}$ +10.2). The aglycone fractions from compounds **1–5** exhibited negative optical rotation values ranging from −6.2 to −9.6, respectively.

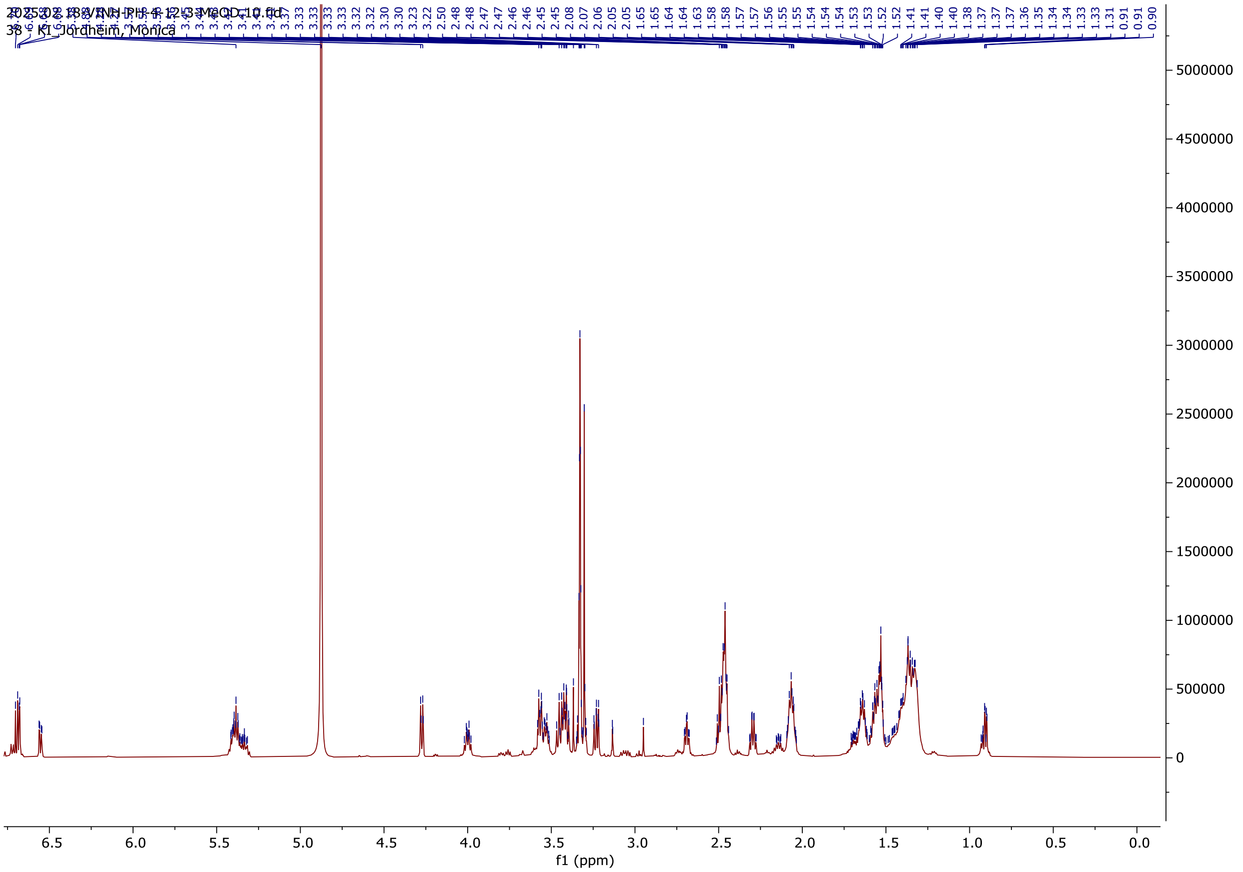

Figure S1: ^{1}H NMR spectrum (CD_3OD, 600 MHz) of compound **1**

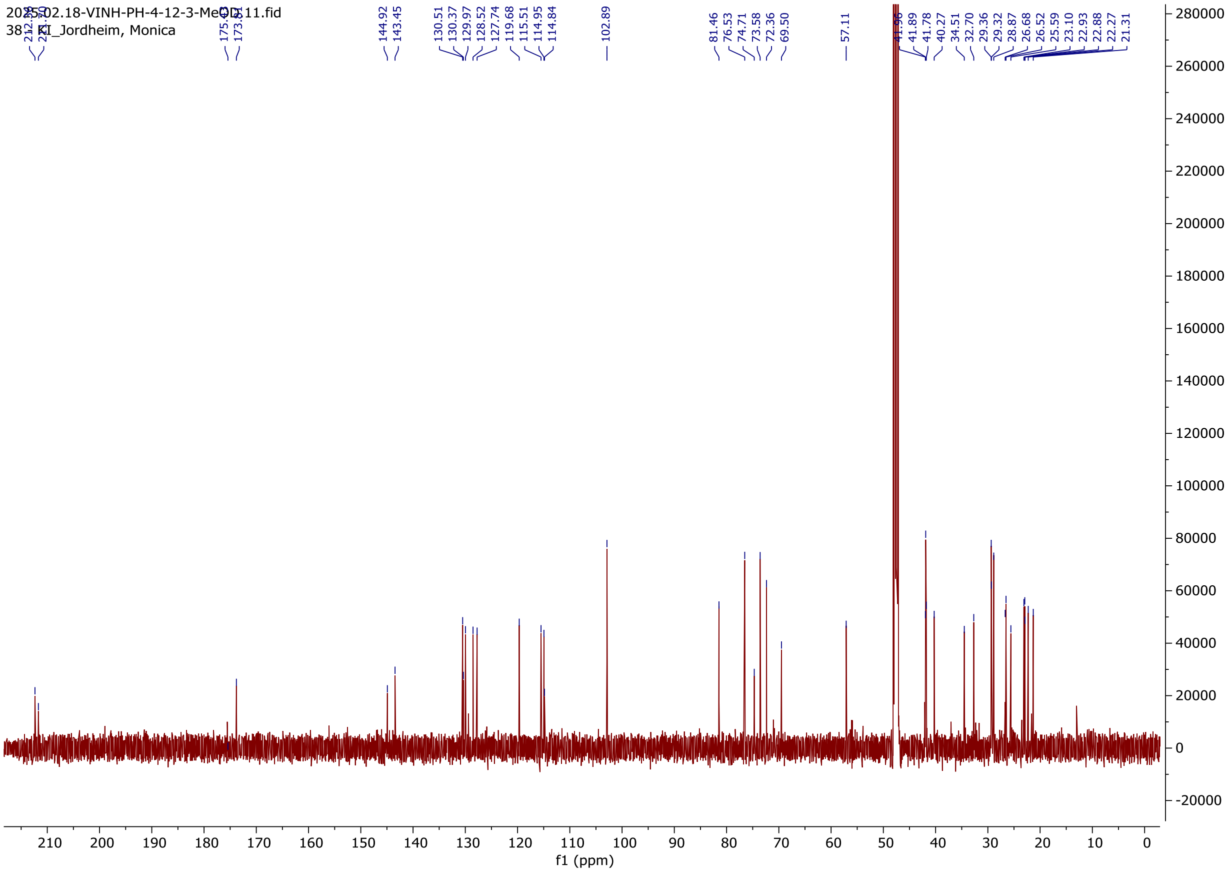

Figure S2: ^{13}C NMR spectrum (CD_3OD, 150 MHz) of compound **1**

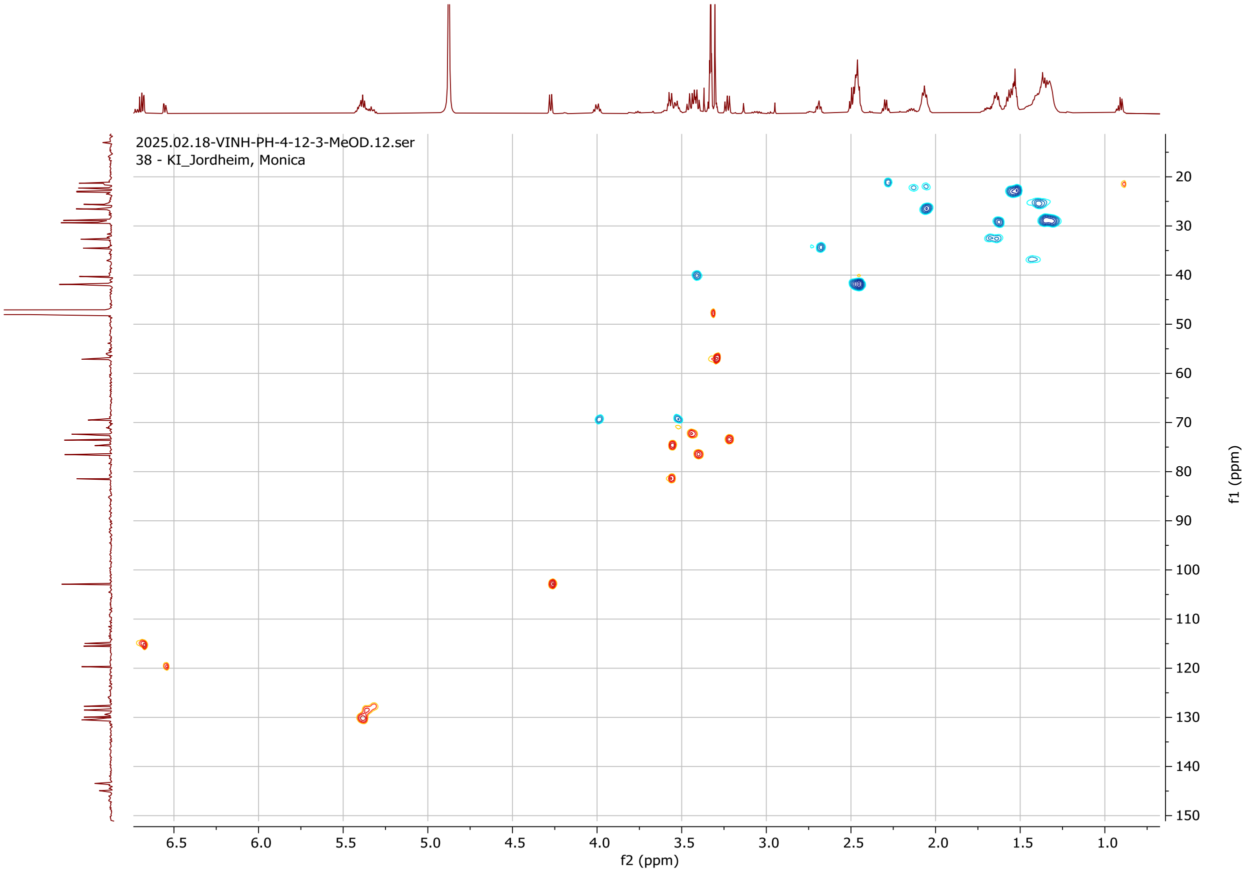

Figure S3: ^1H-^{13}C HSQC spectrum (CD_3OD, 600 MHz) of compound **1**

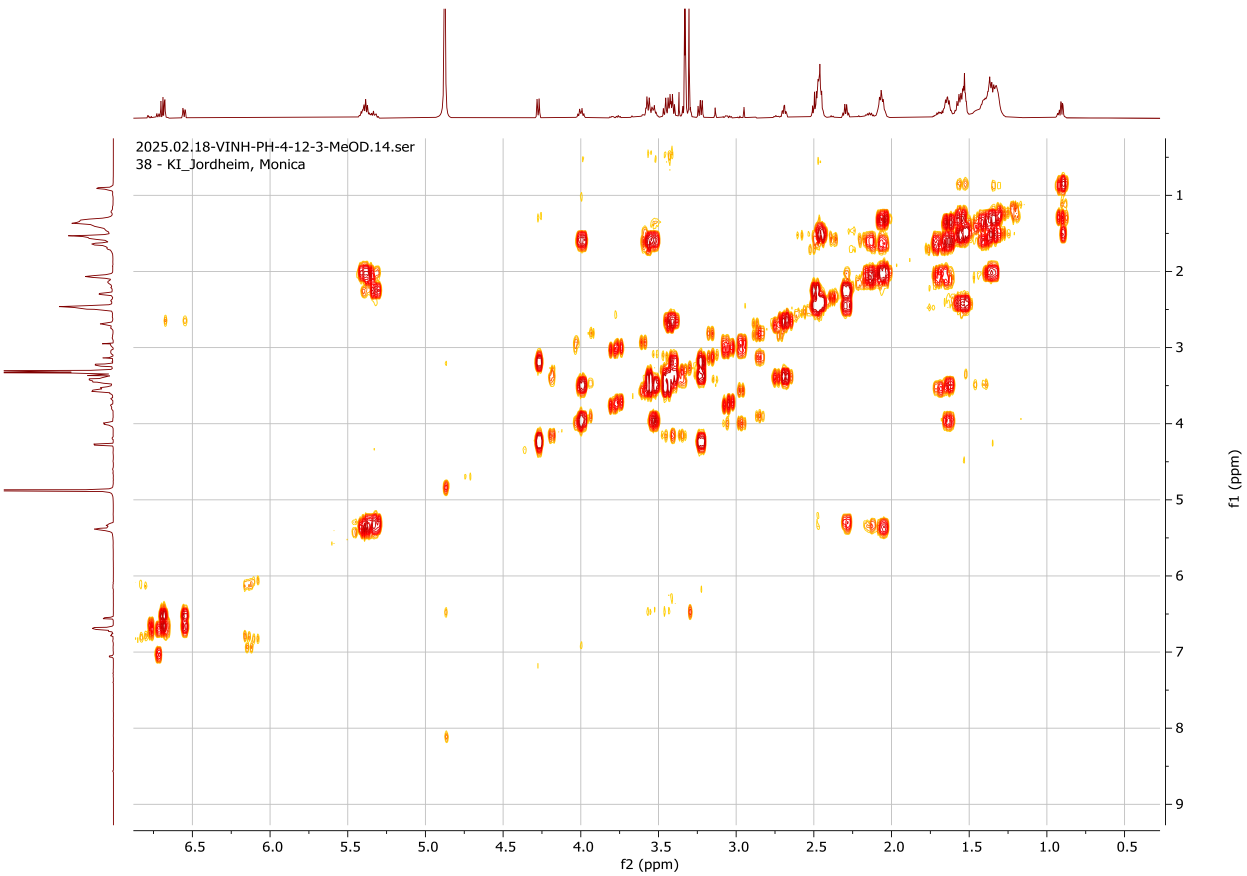

Figure S4: ¹H-¹H COSY spectrum (CD_3OD, 600 MHz) of compound **1**

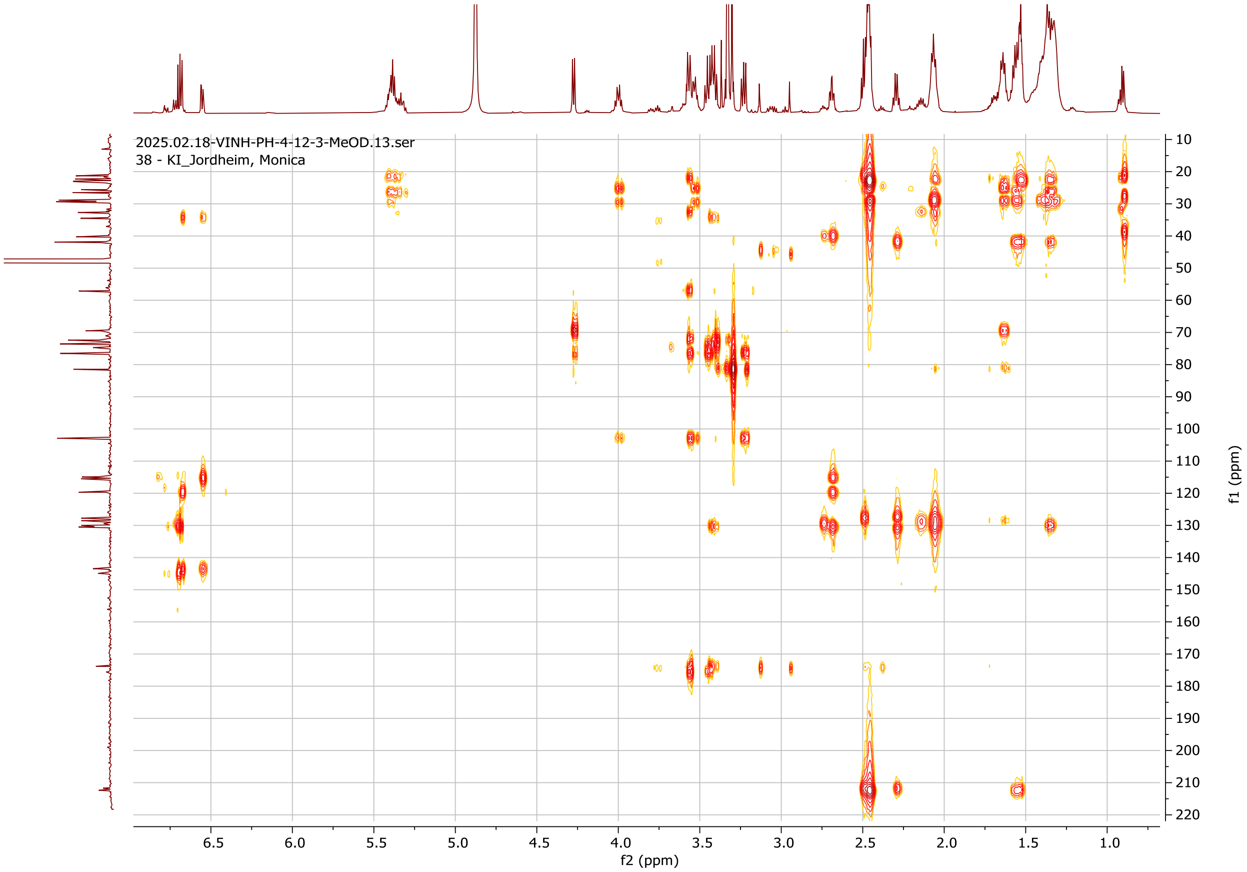

Figure S5: ^{1}H-^{13}C HMBC spectrum (CD_3OD, 600 MHz) of compound **1**

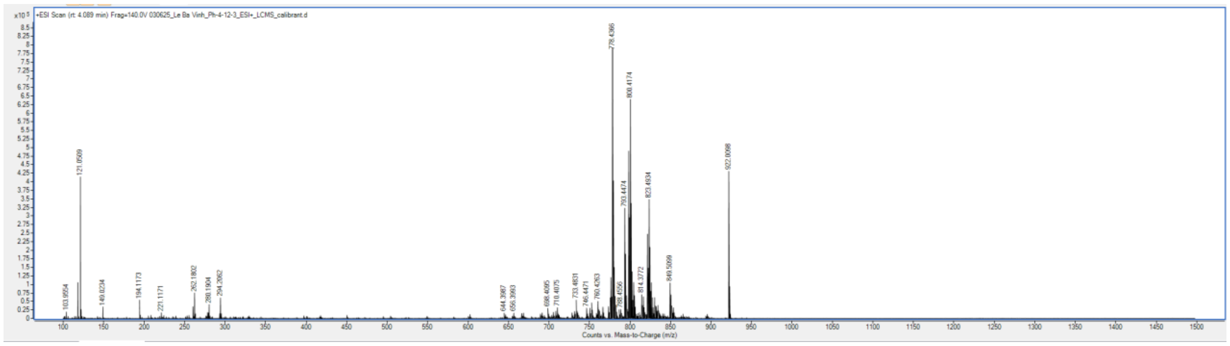

Figure S6: HR-ESI-MS spectrum of compound **1**

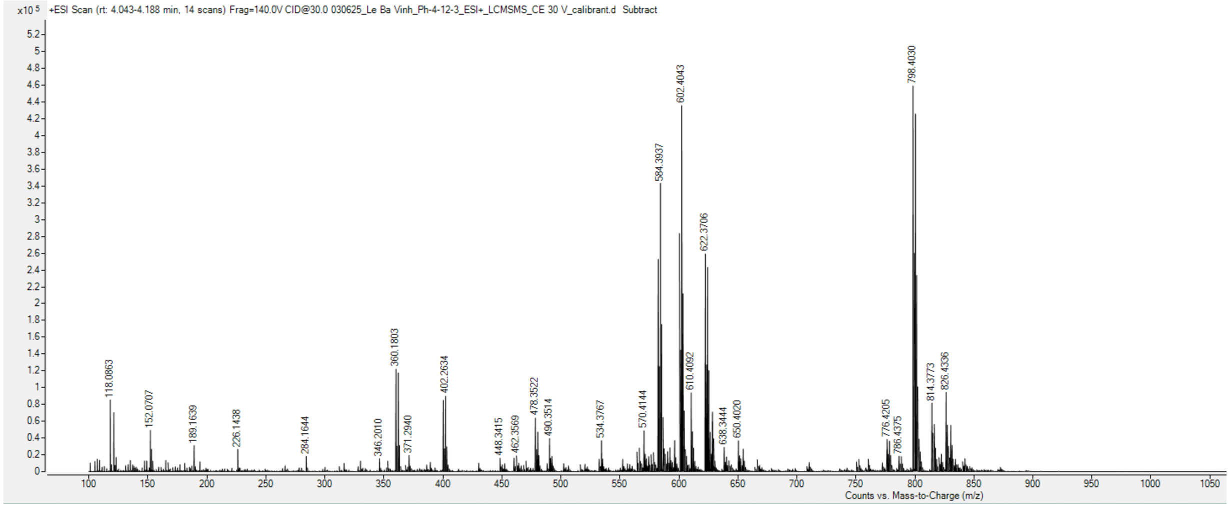

Figure S7: HR-ESI-MS/MS spectrum of compound **1**

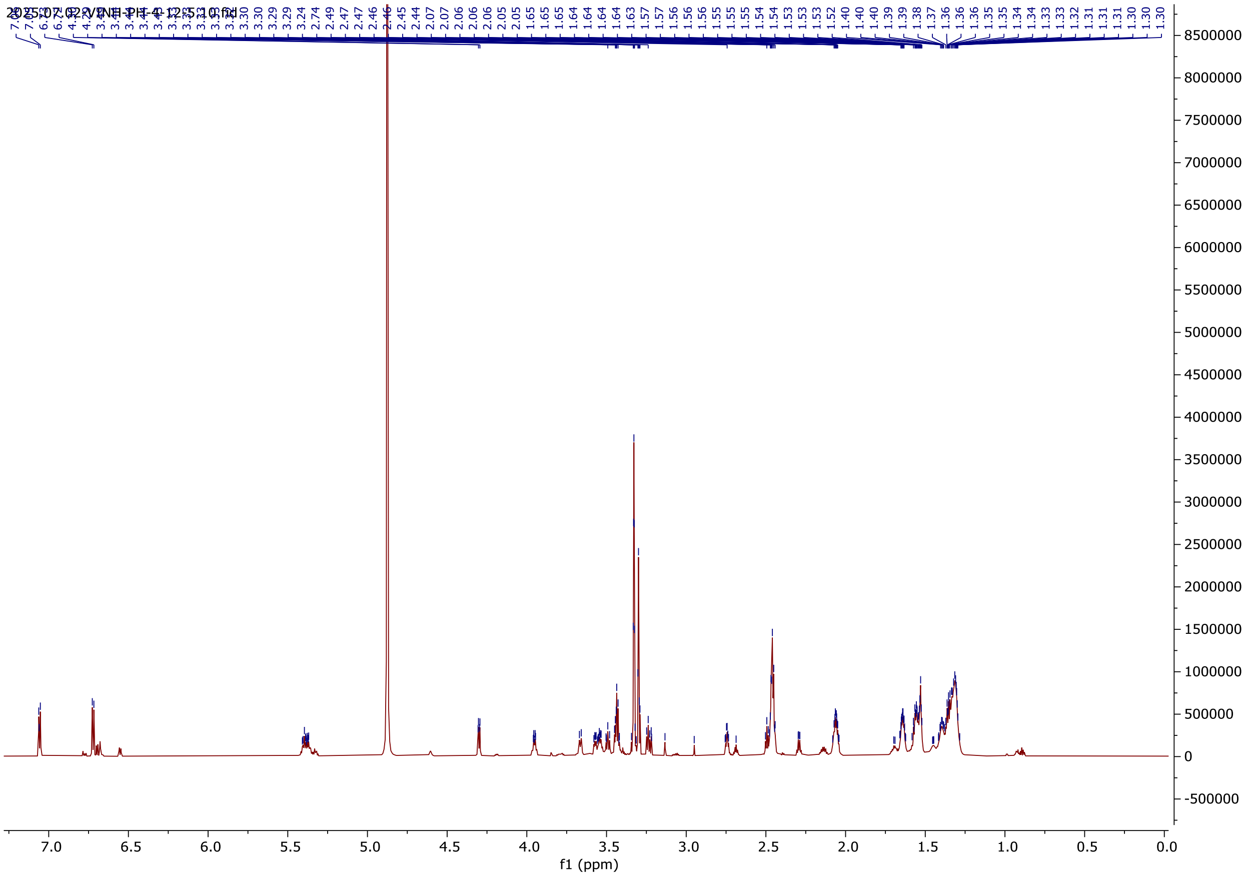

Figure S8: ^{1}H NMR spectrum (CD_3OD, 600 MHz) of compound **2**

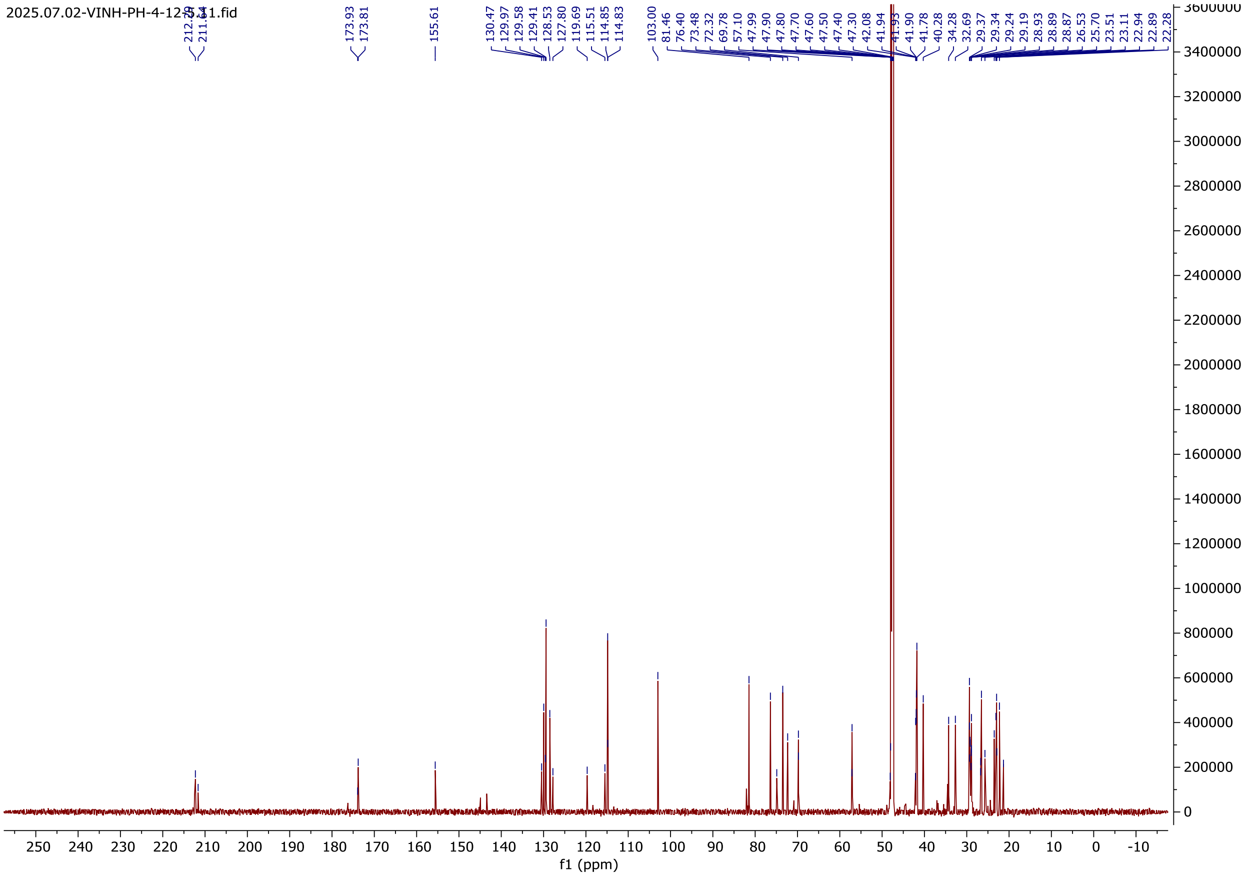

Figure S9: ^{13}C NMR spectrum (CD_3OD, 150 MHz) of compound **2**

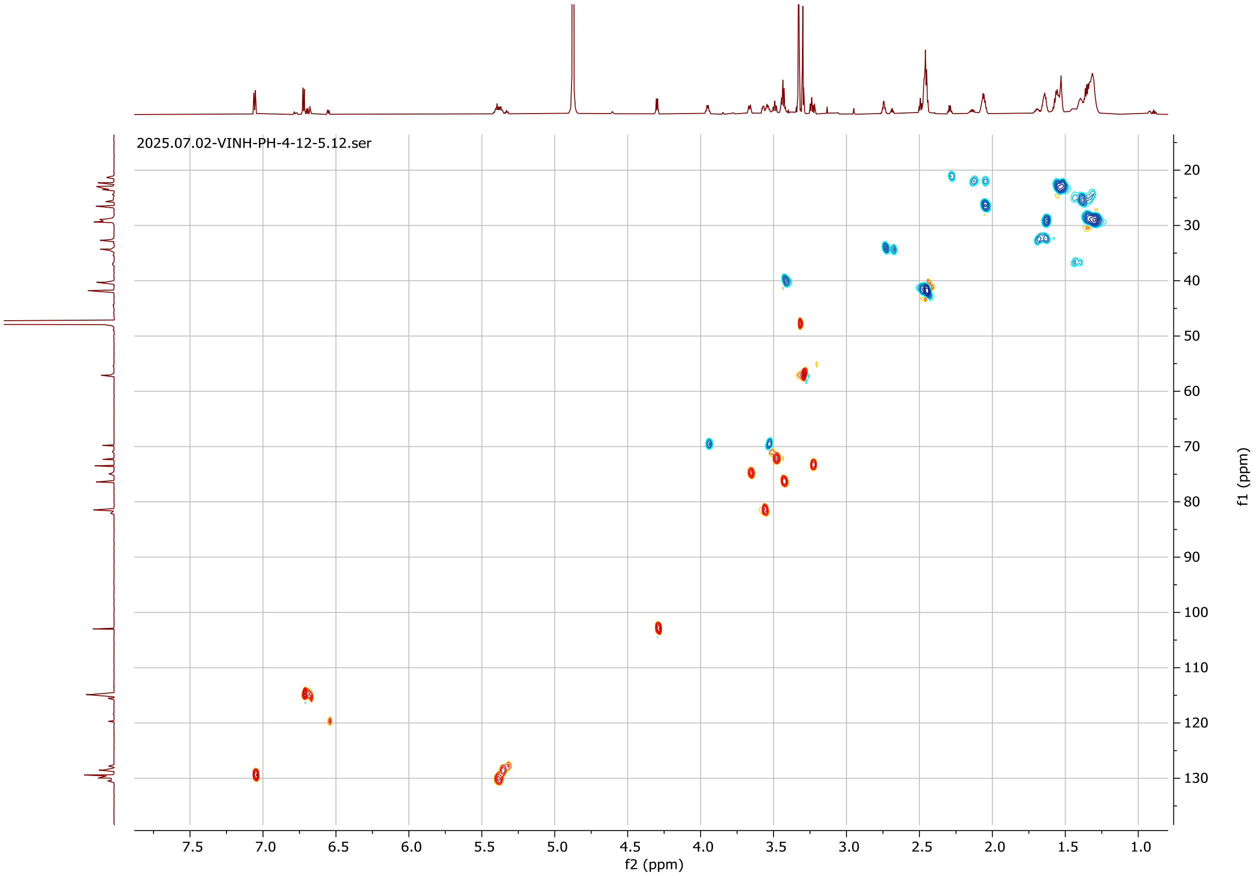

Figure S10: ^1H-^{13}C HSQC spectrum (CD_3OD, 600 MHz) of compound **2**

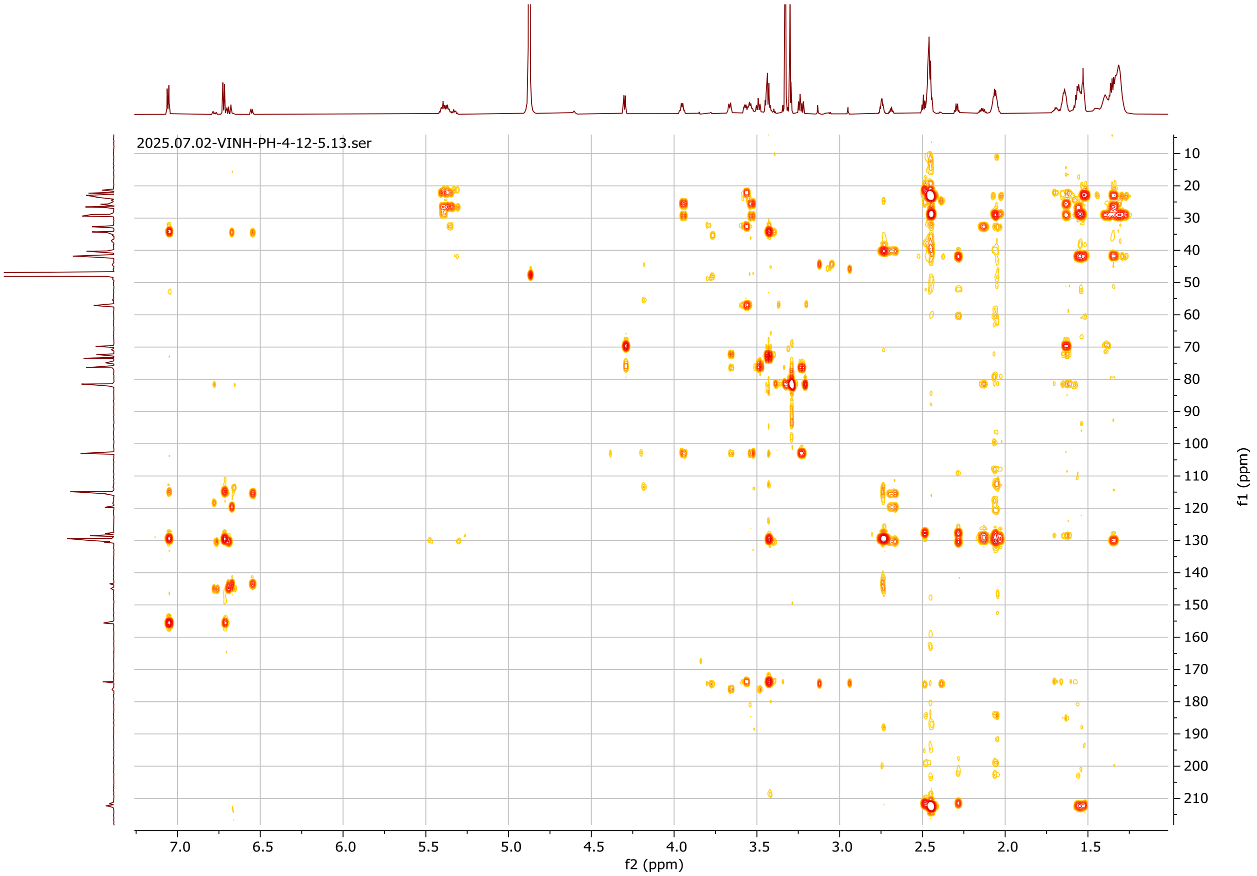

Figure S11: ^{1}H-^{13}C HMBC spectrum (CD_3OD, 600 MHz) of compound **2**

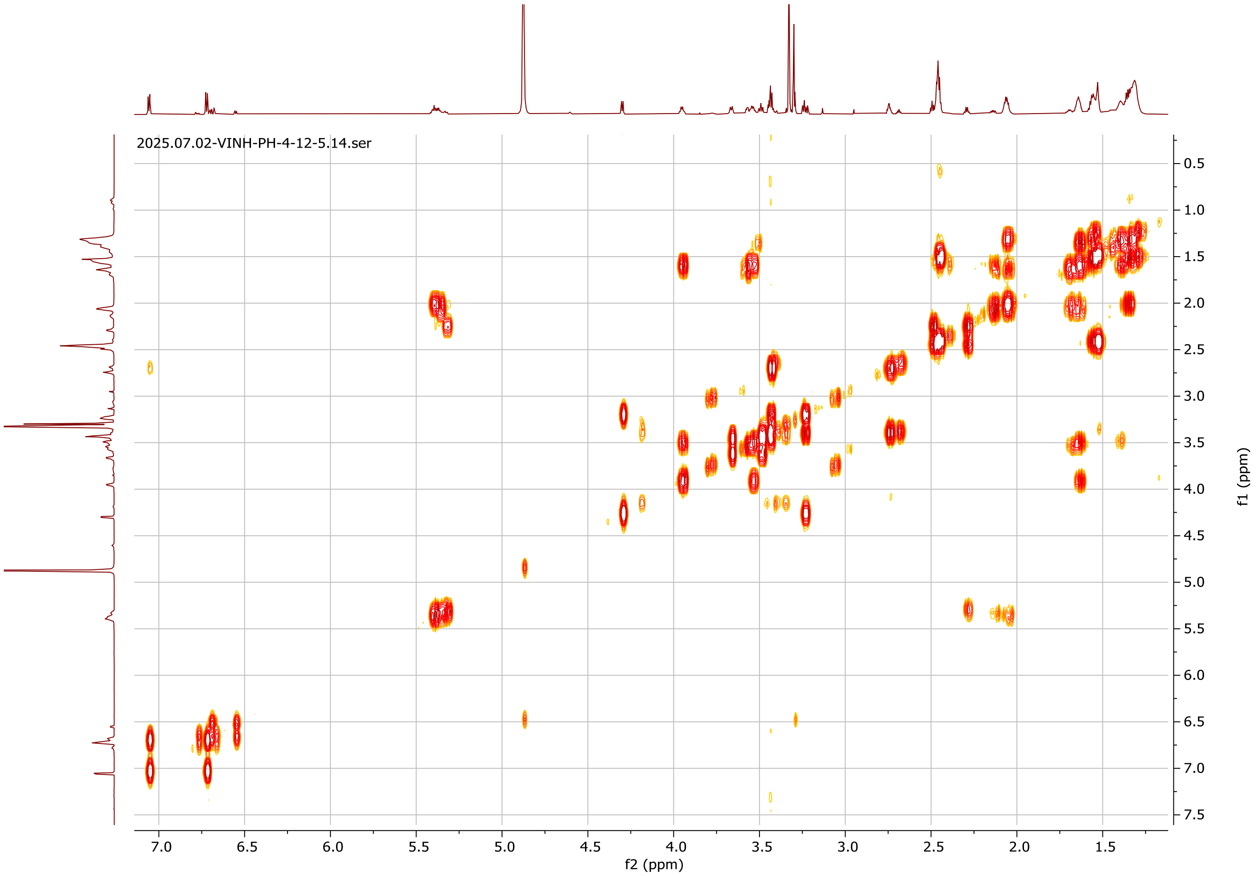

Figure S12: ^{1}H-^{1}H COSY spectrum (CD_3OD, 600 MHz) of compound **2**

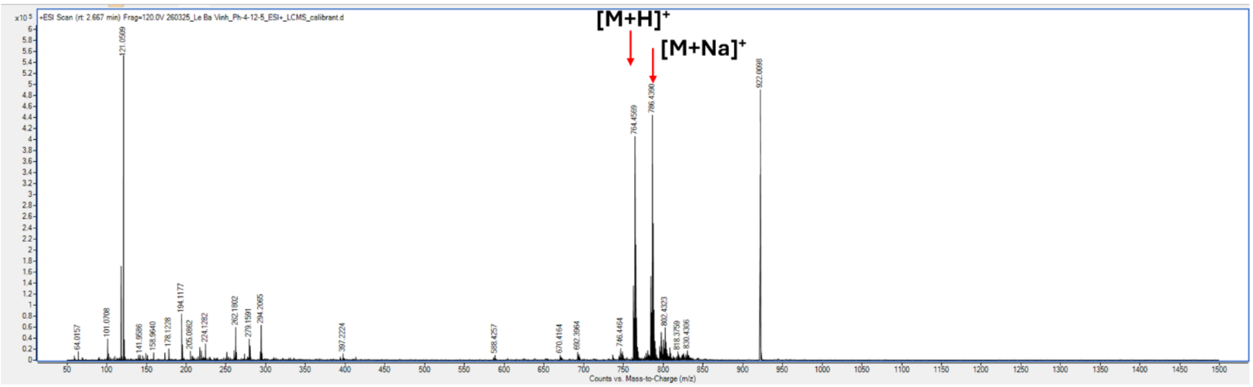

Figure S13: HR-ESI-MS spectrum of compound **2**

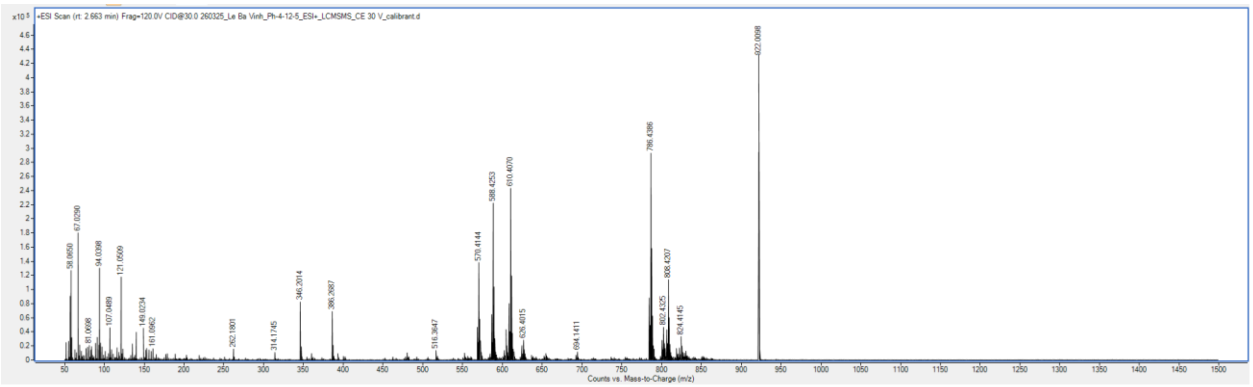

Figure S14: HR-ESI-MS/MS spectrum of compound **2**

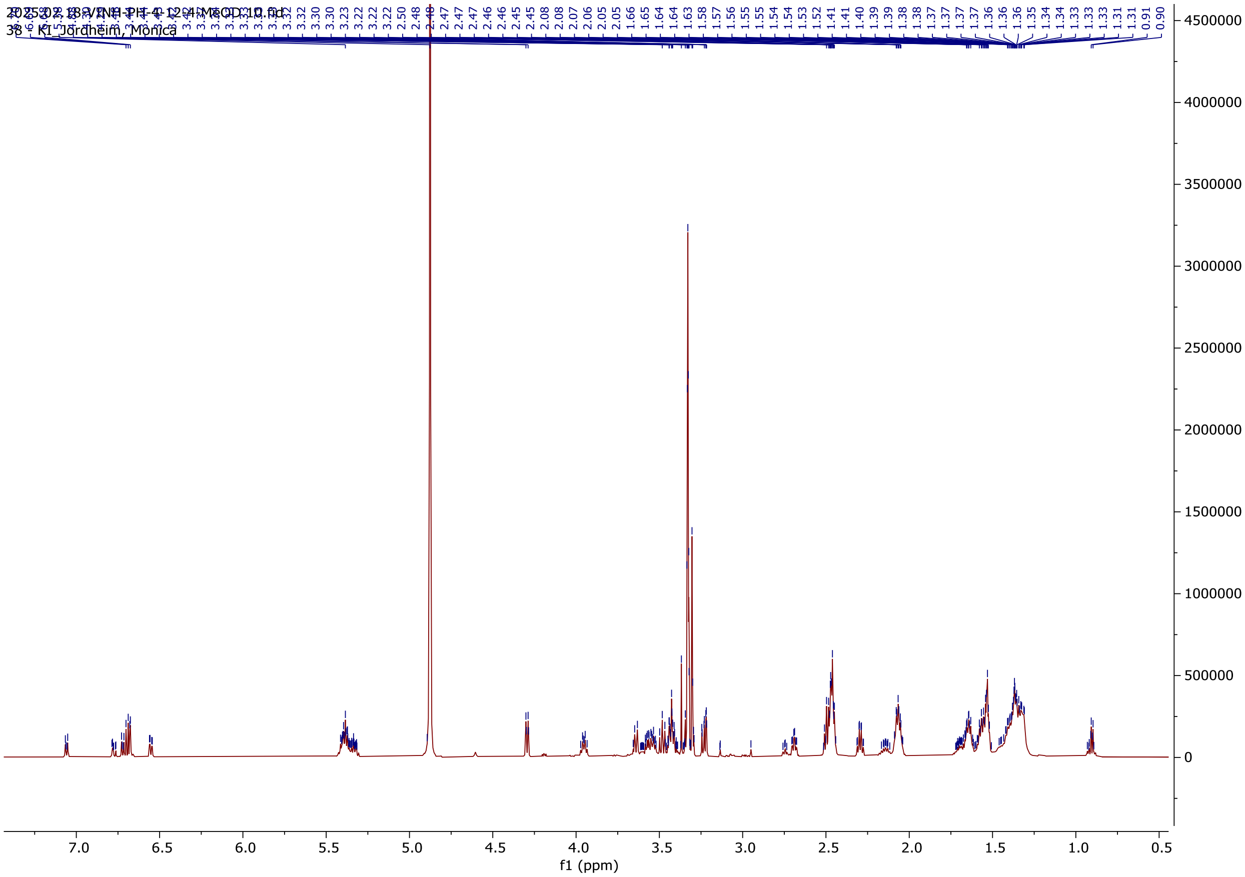

Figure S15: ^{1}H NMR spectrum (CD_3OD, 600 MHz) of compound **3**

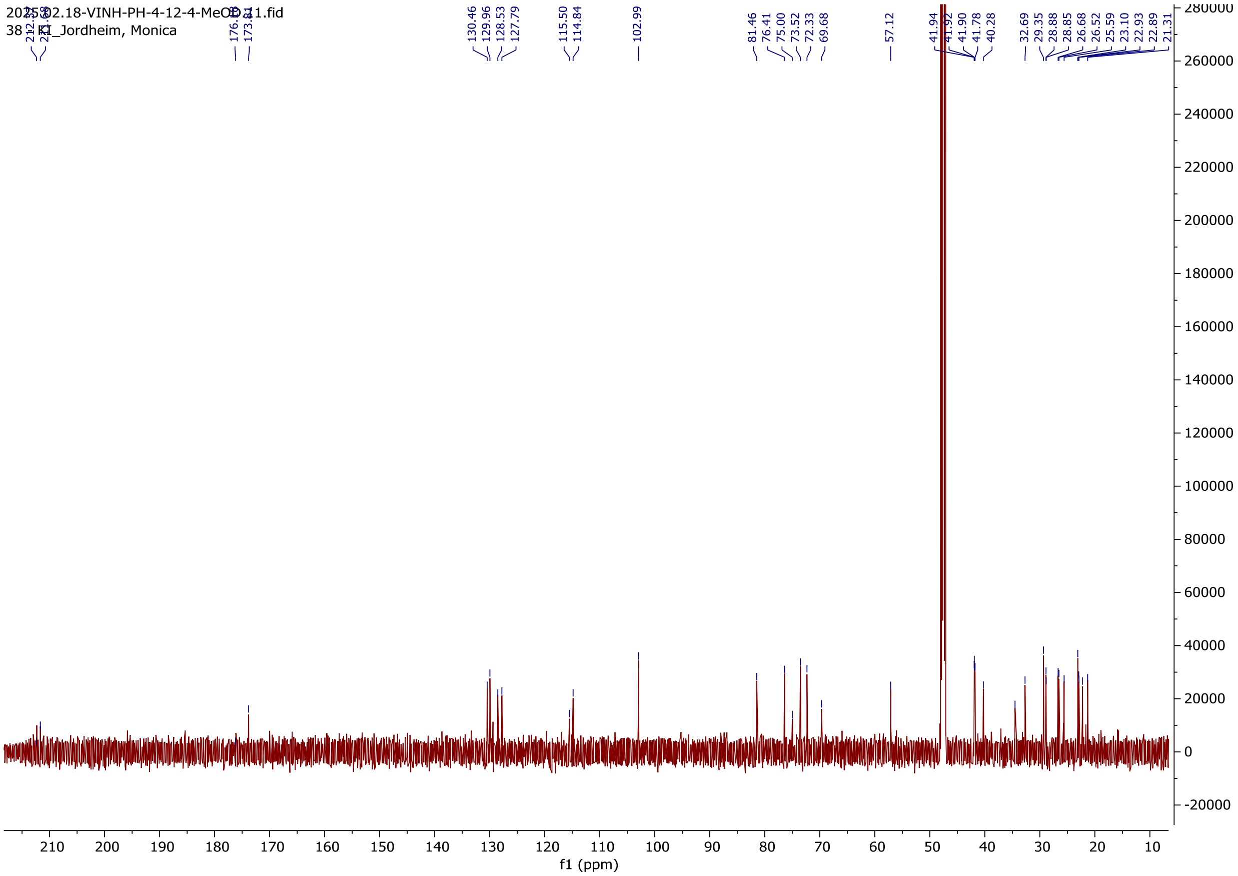

Figure S16: ^{13}C NMR spectrum (CD_3OD, 150 MHz) of compound **3**

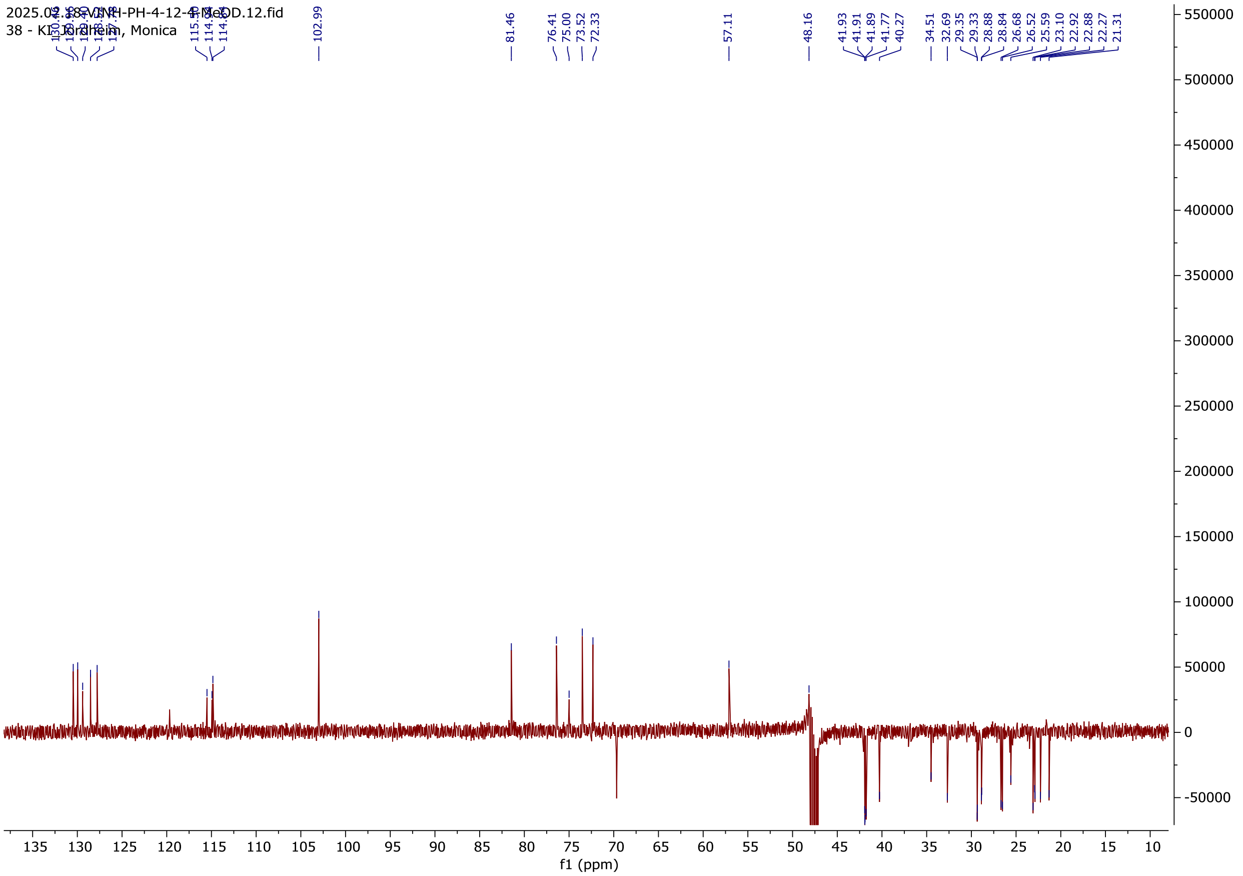

Figure S17: DEPT spectrum (CD_3OD, 150 MHz) of compound **3**

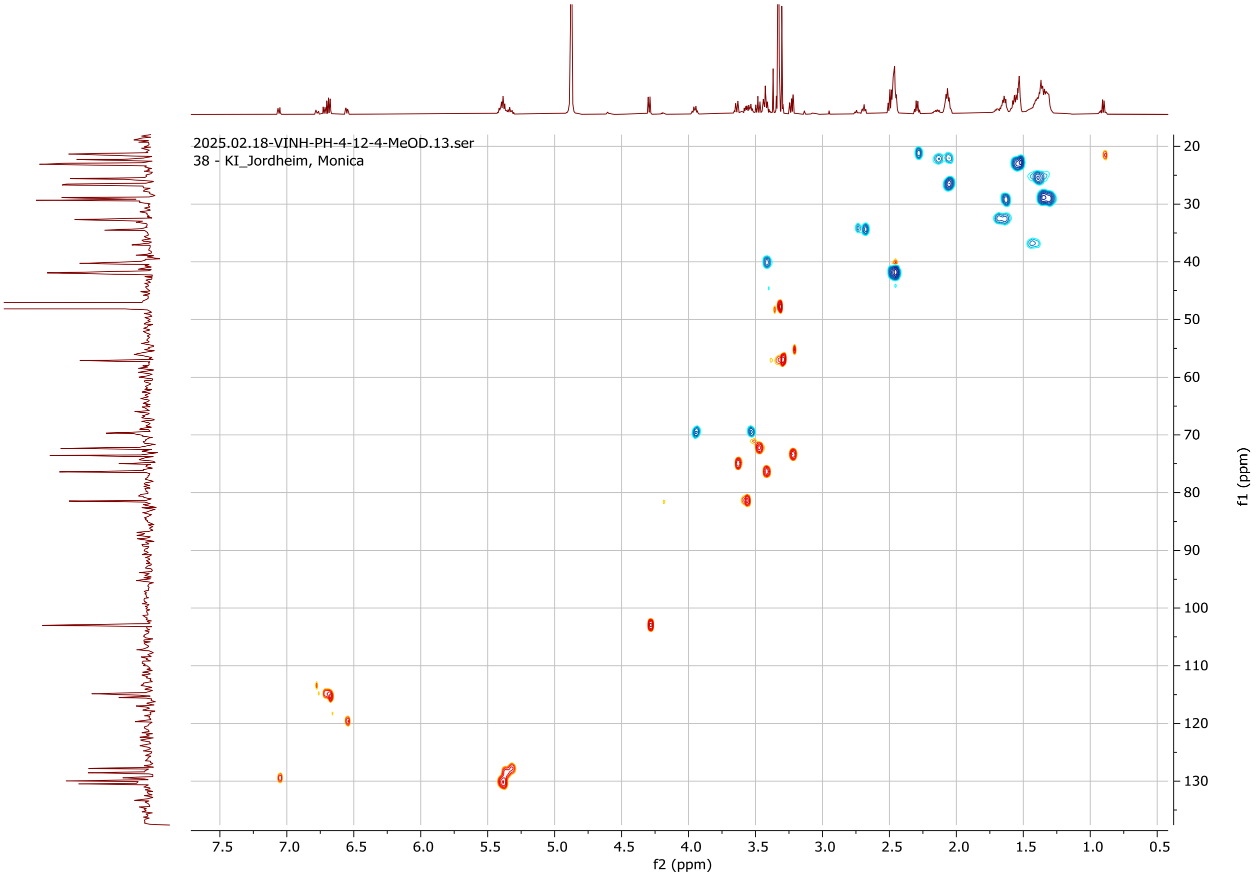

Figure S18: ^{1}H-^{13}C HSQC spectrum (CD_3OD, 600 MHz) of compound **3**

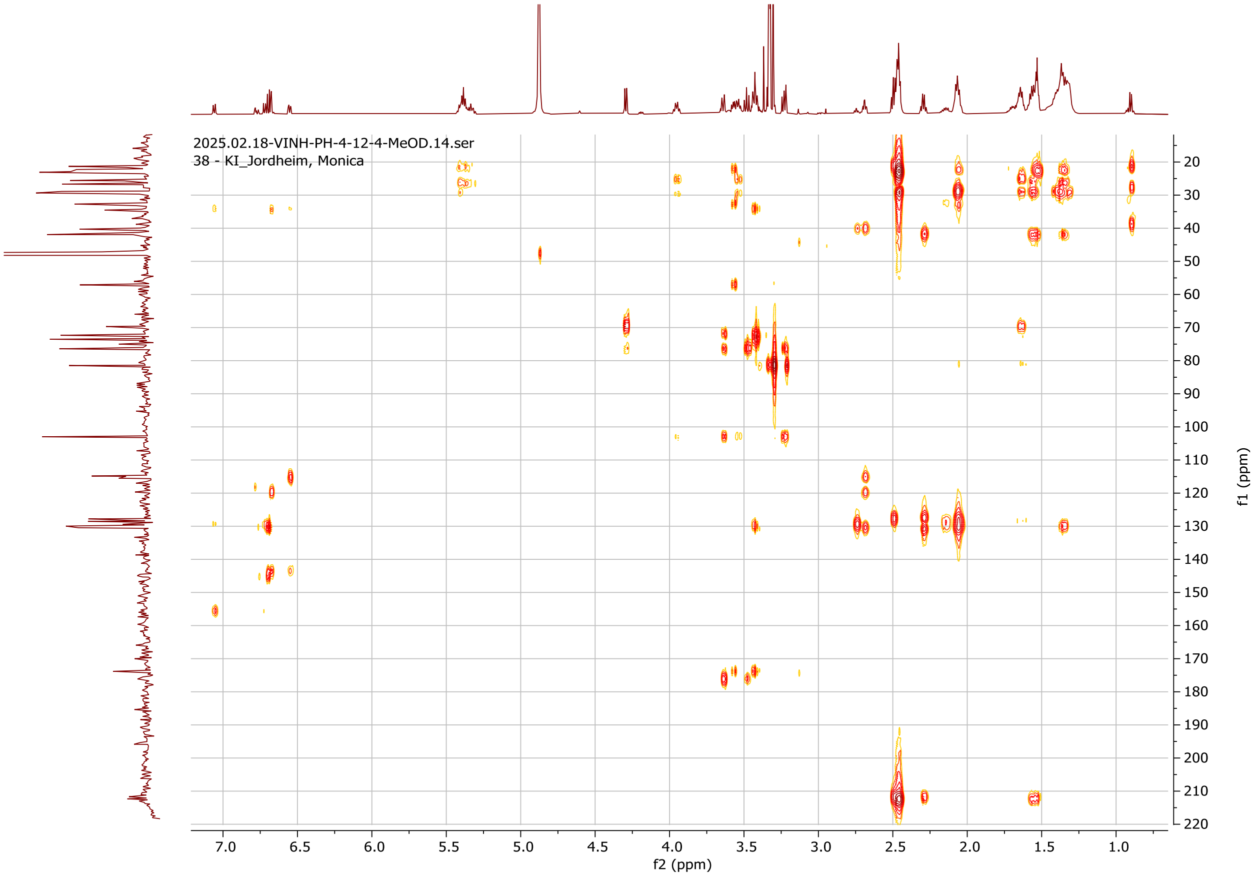

Figure S19: ^{1}H-^{13}C HMBC spectrum (CD_3OD, 600 MHz) of compound **3**

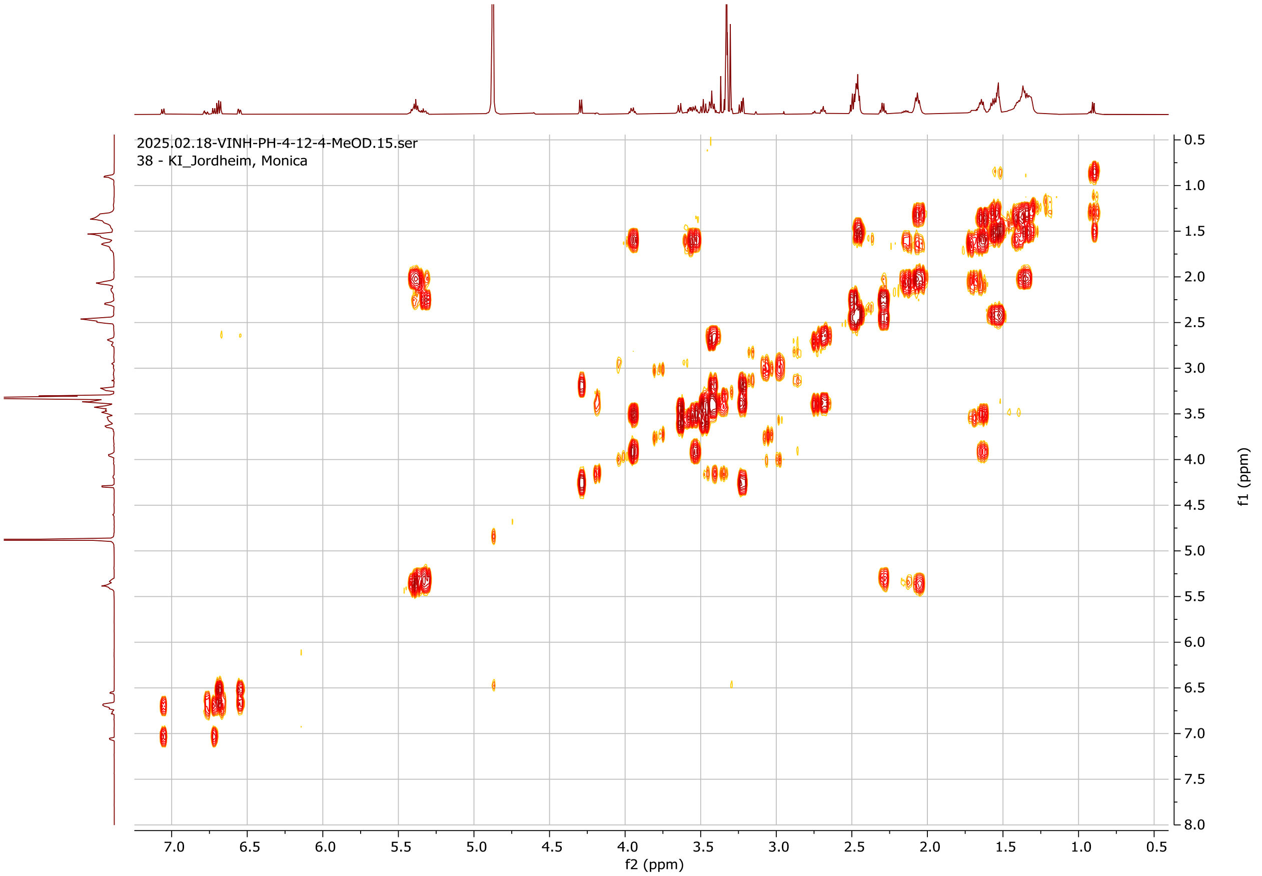

Figure S20: ^{1}H-^{1}H COSY spectrum (CD_3OD, 600 MHz) of compound **3**

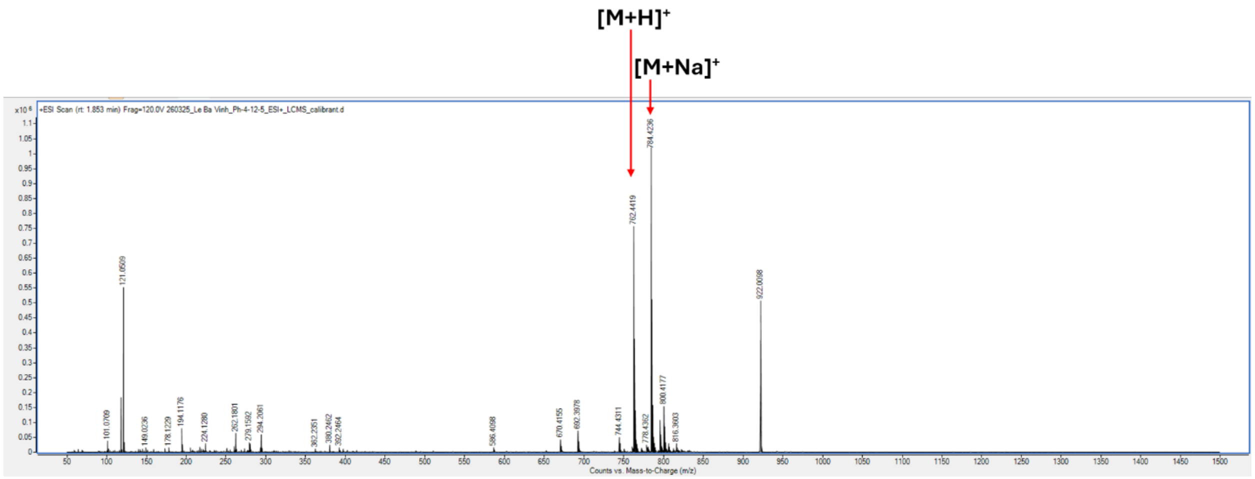

Figure S21: HR-ESI-MS spectrum of compound **3**

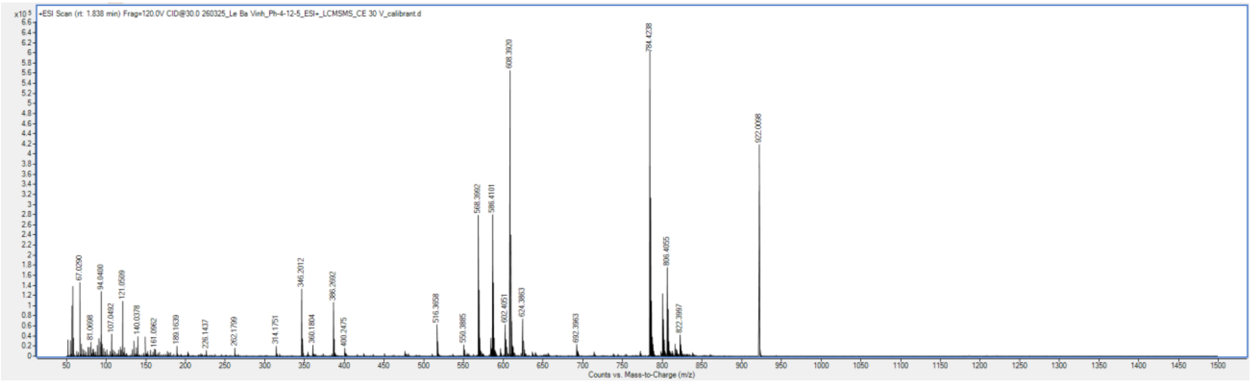

Figure S22: HR-ESI-MS/MS spectrum of compound **3**

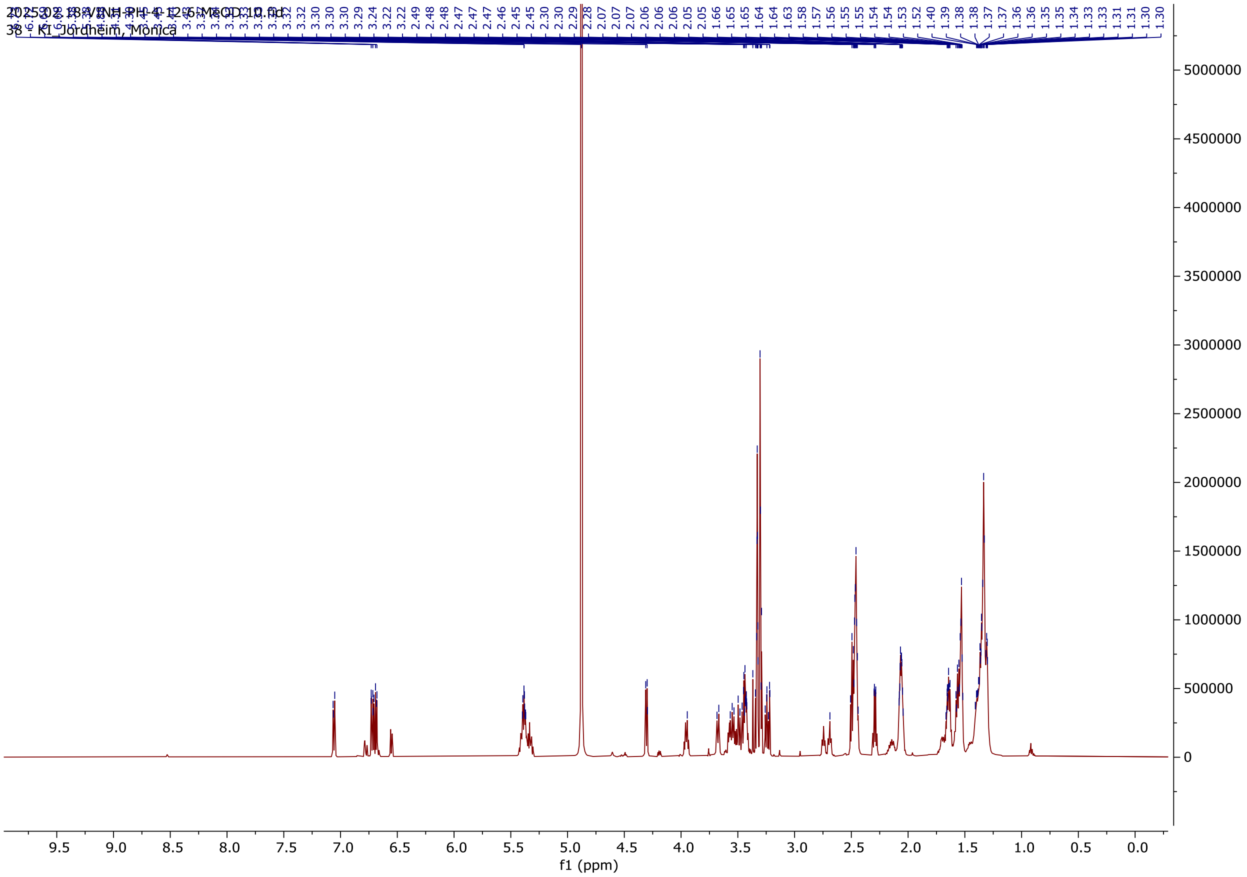

Figure S23: ^{1}H NMR spectrum (CD_3OD, 600 MHz) of compound **4**

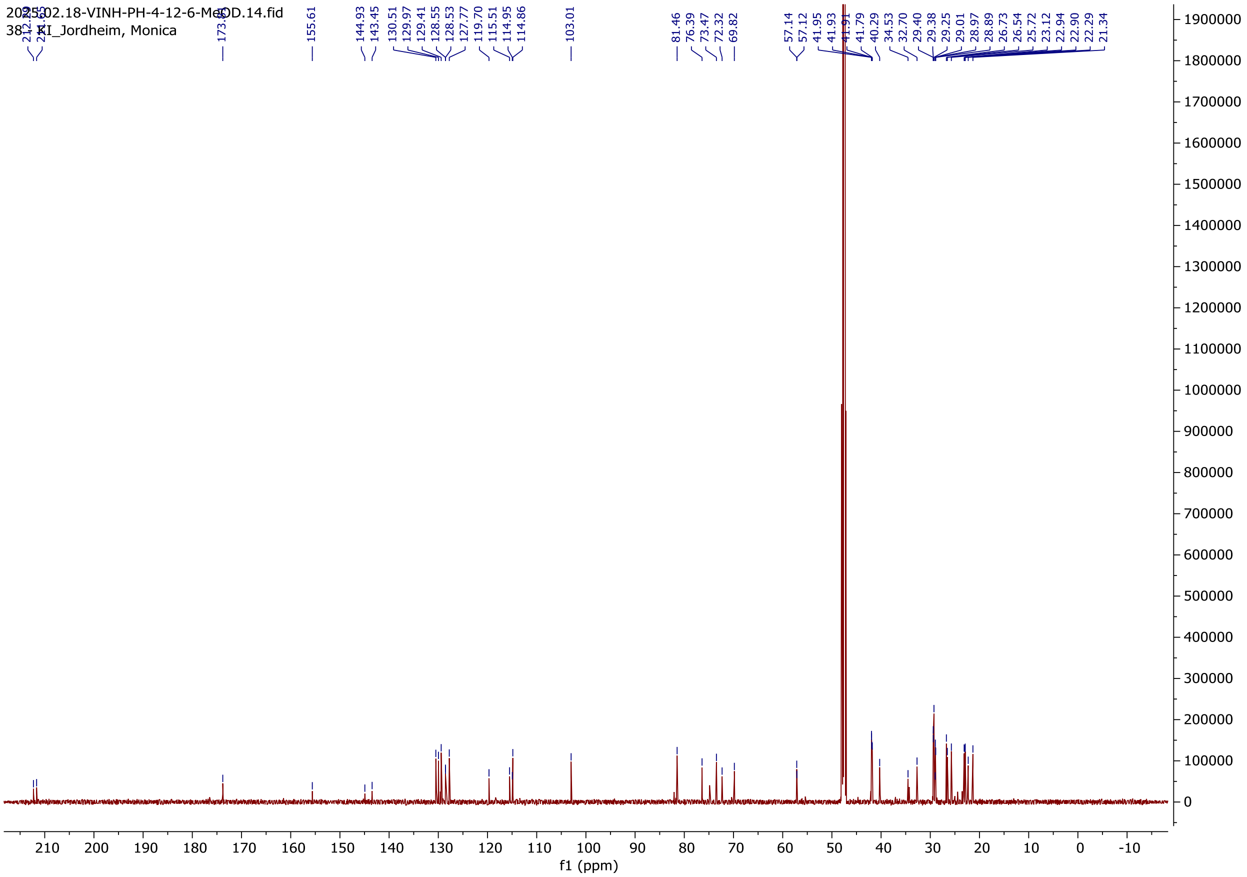

Figure S24: ^{13}C NMR spectrum (CD_3OD, 150 MHz) of compound **4**

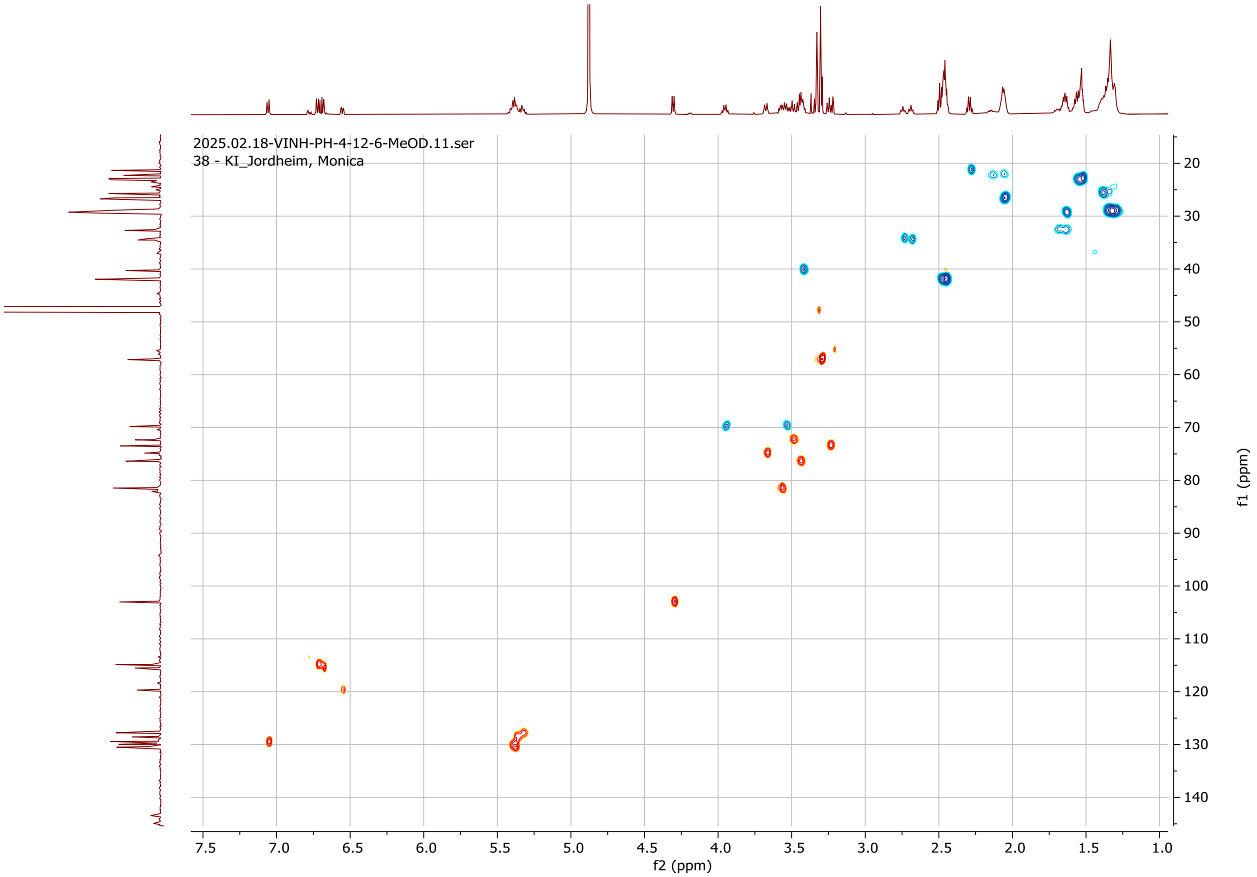

Figure S25: ^{1}H-^{13}C HSQC spectrum (CD_3OD, 600 MHz) of compound **4**

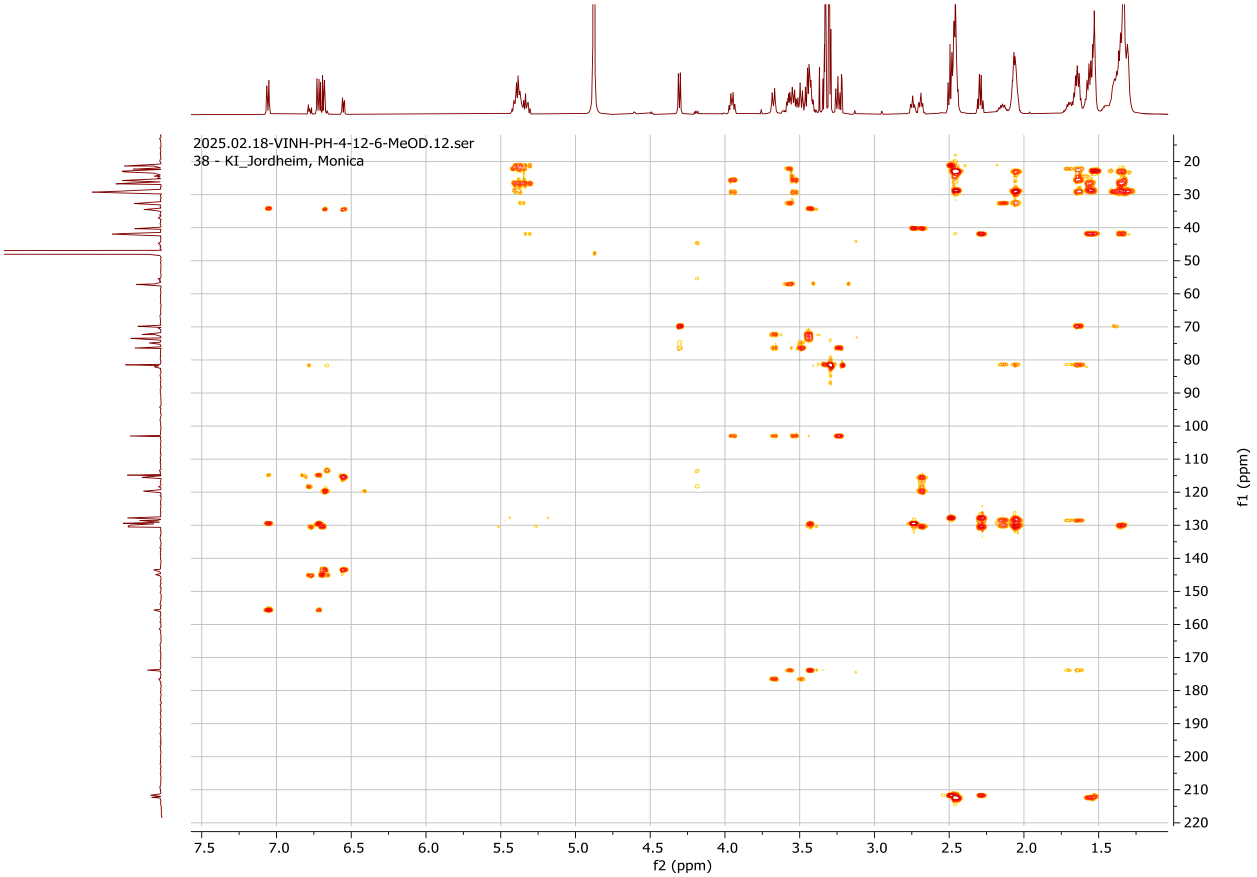

Figure S26: ^1H-^{13}C HMBC spectrum (CD_3OD, 600 MHz) of compound **4**

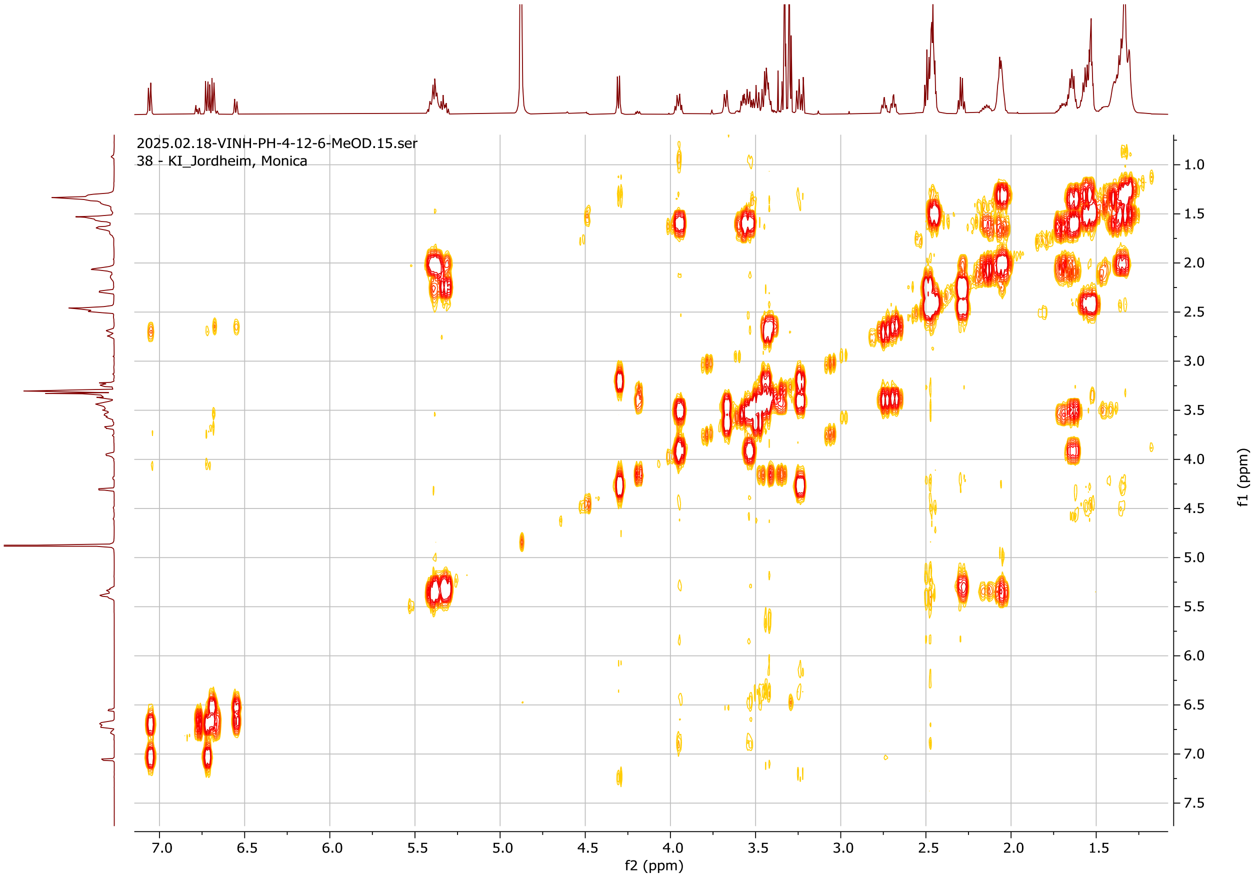

Figure S27: ^1H-^1H COSY spectrum (CD_3OD, 600 MHz) of compound **4**

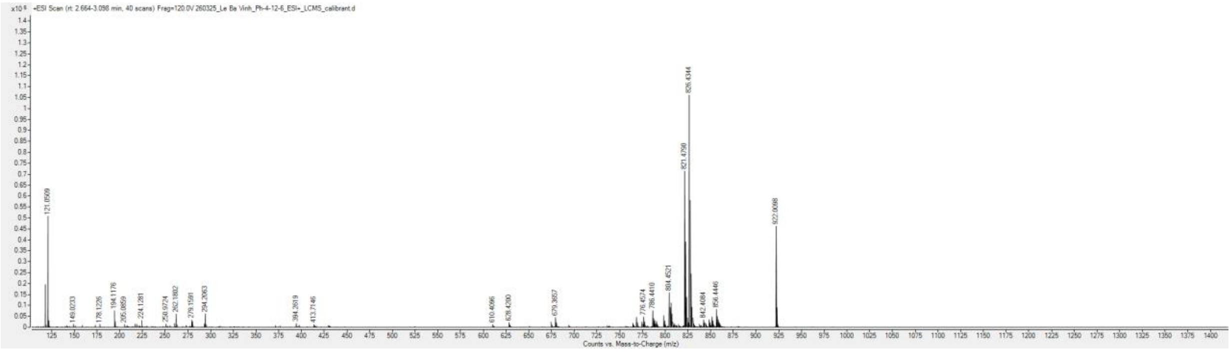

Figure S28: HR-ESI-MS spectrum of compound **4**

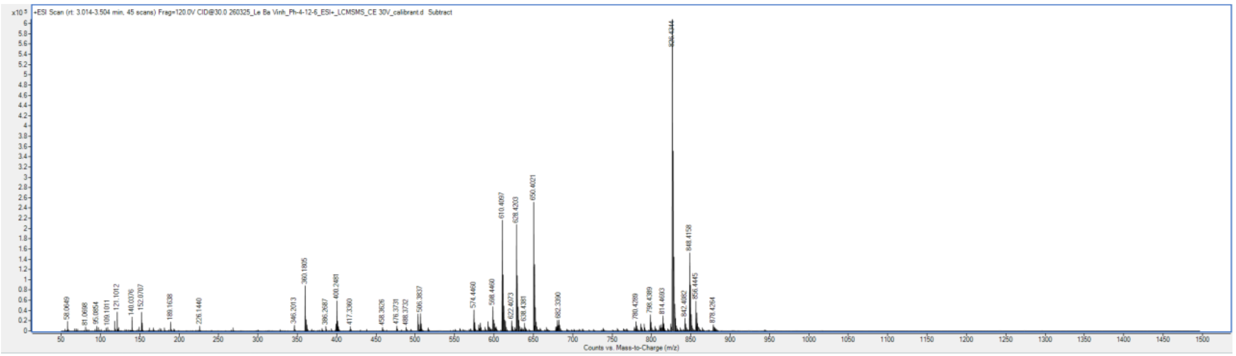

Figure S29: HR-ESI-MS/MS spectrum of compound **4**

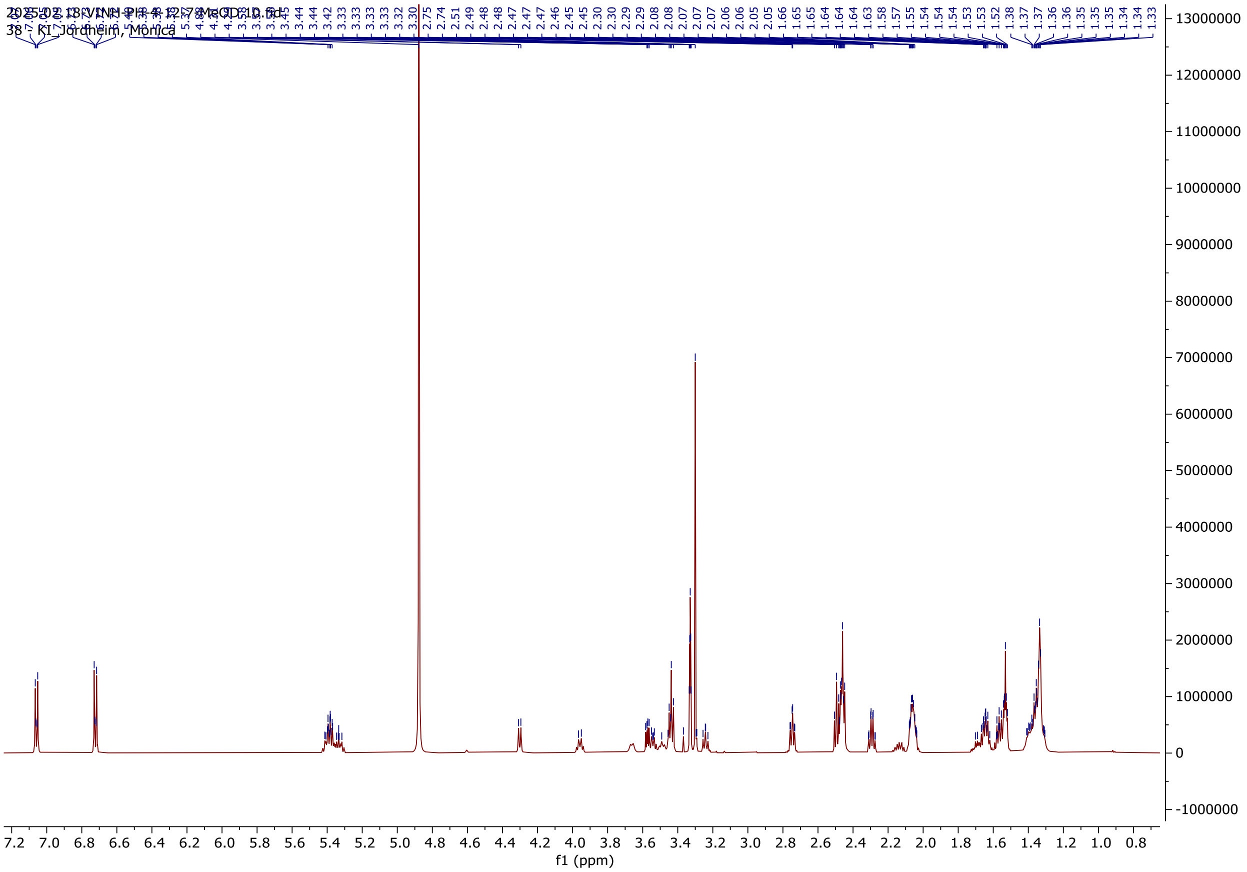

Figure S30: ^{1}H NMR spectrum (CD_3OD, 600 MHz) of compound **5**

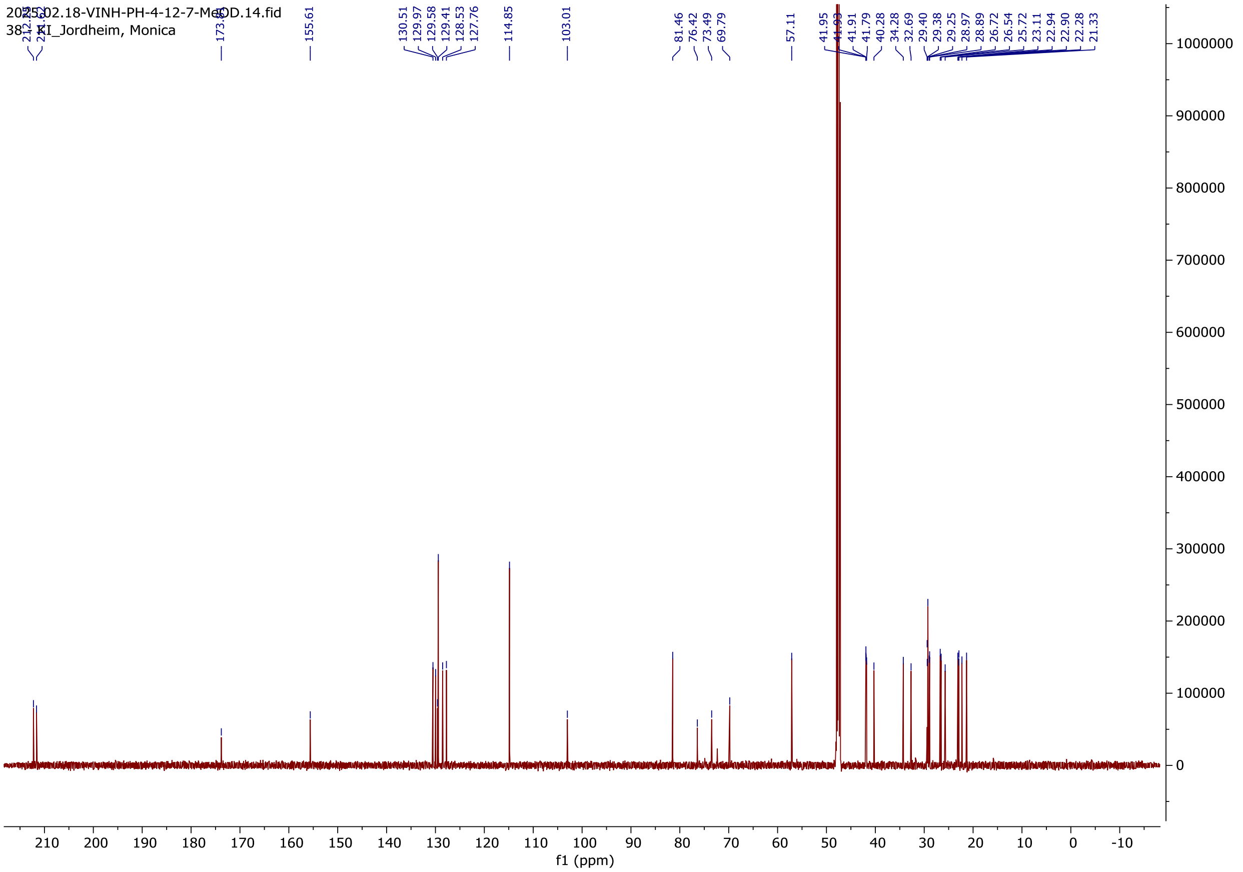

Figure S31: ^{13}C NMR spectrum (CD_3OD, 150 MHz) of compound **5**

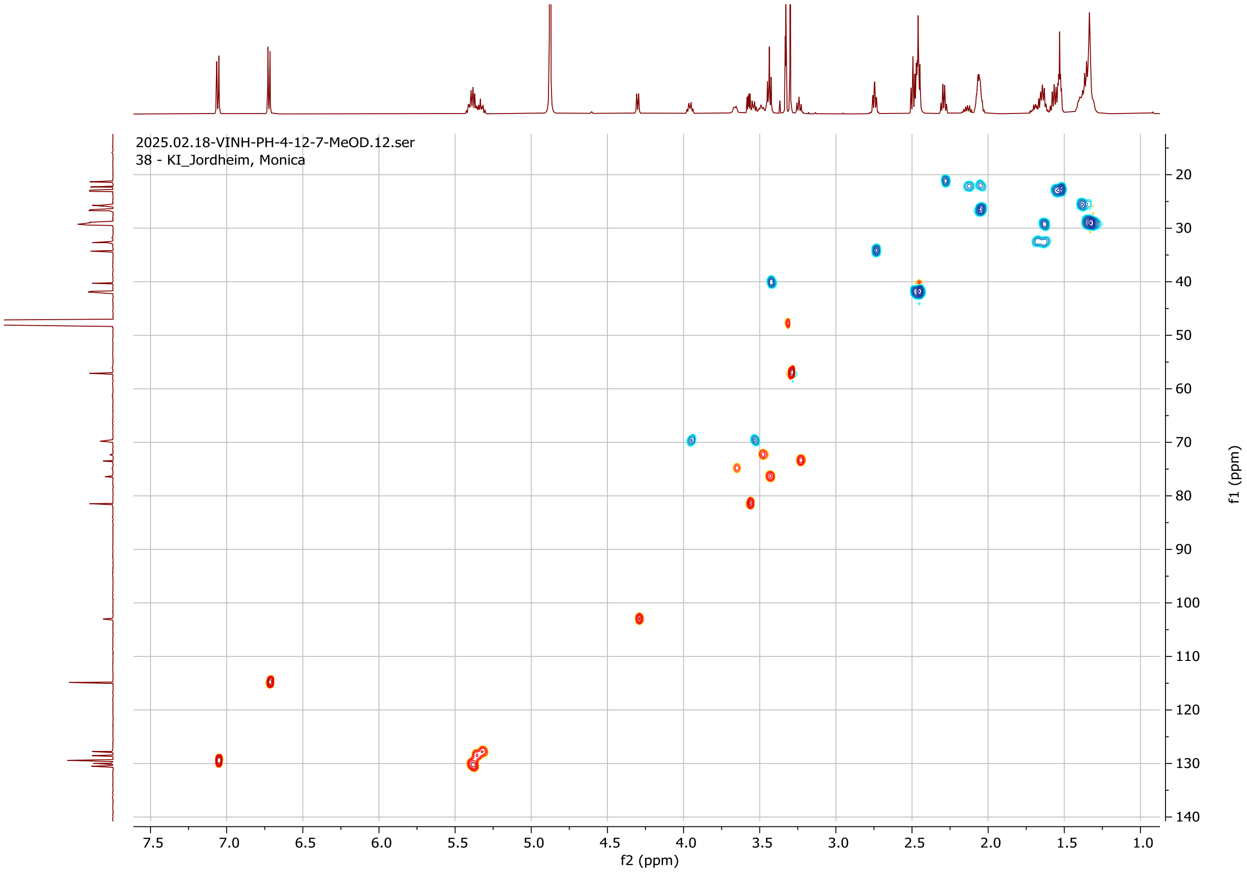

Figure S32: ^{1}H-^{13}C HSQC spectrum (CD_3OD, 600 MHz) of compound **5**

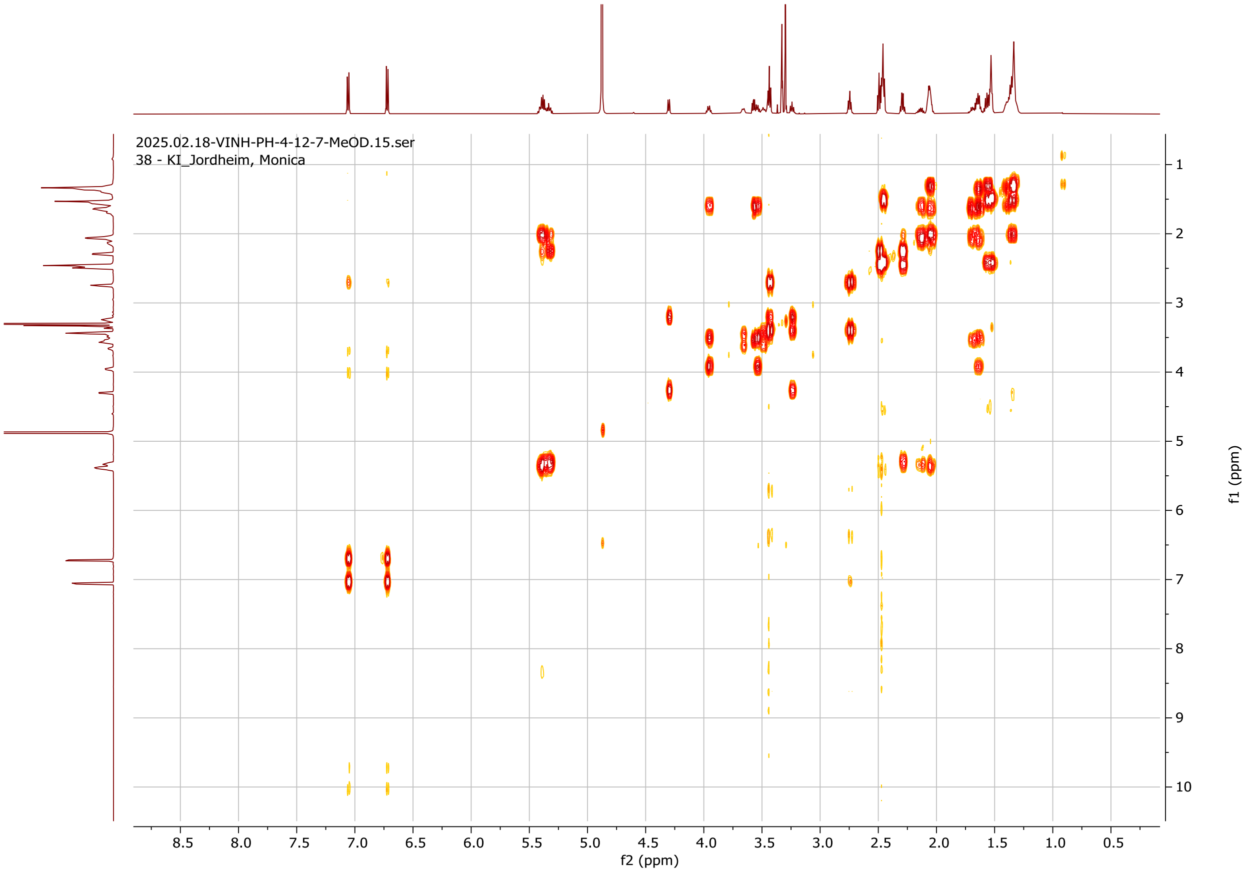

Figure S33: ^1H-^1H COSY spectrum (CD_3OD, 600 MHz) of compound **5**

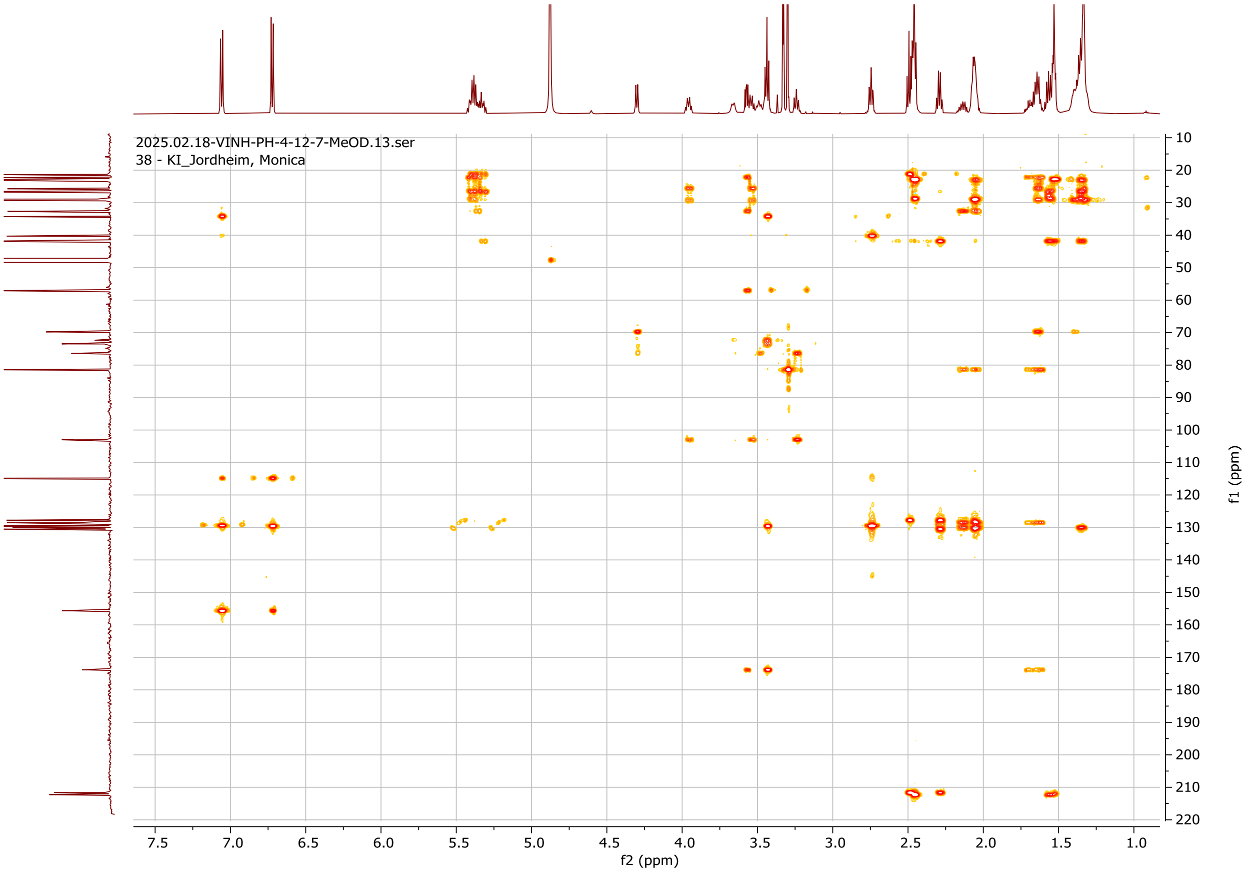

Figure S34: ^{1}H-^{13}C HMBC spectrum (CD_3OD, 600 MHz) of compound **5**

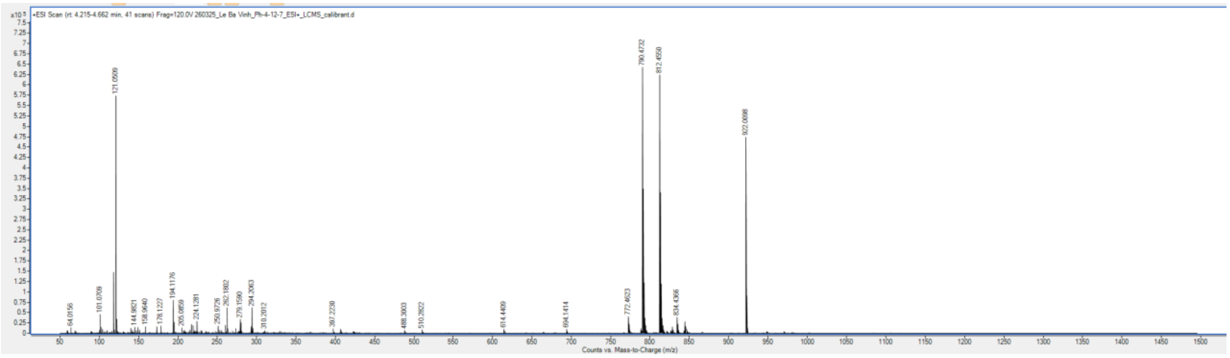

Figure S35: HR-ESI-MS spectrum of compound **5**

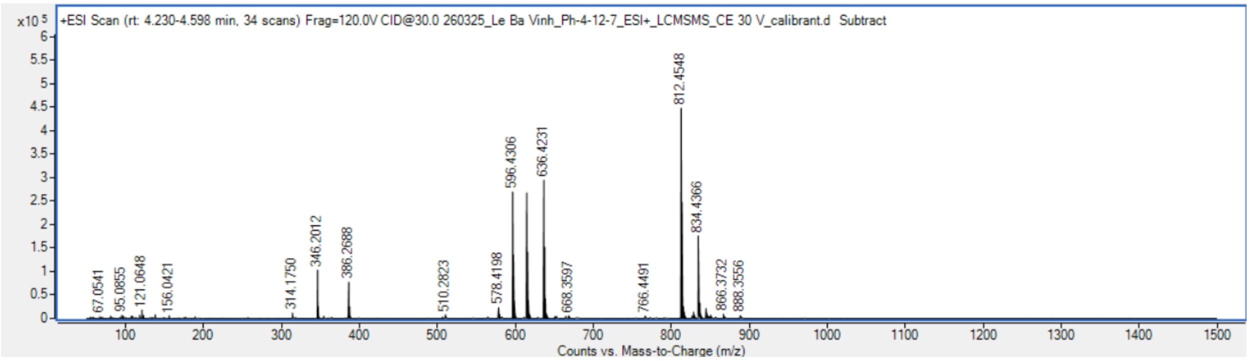

Figure S36: HR-ESI-MS/MS spectrum of compound **5**

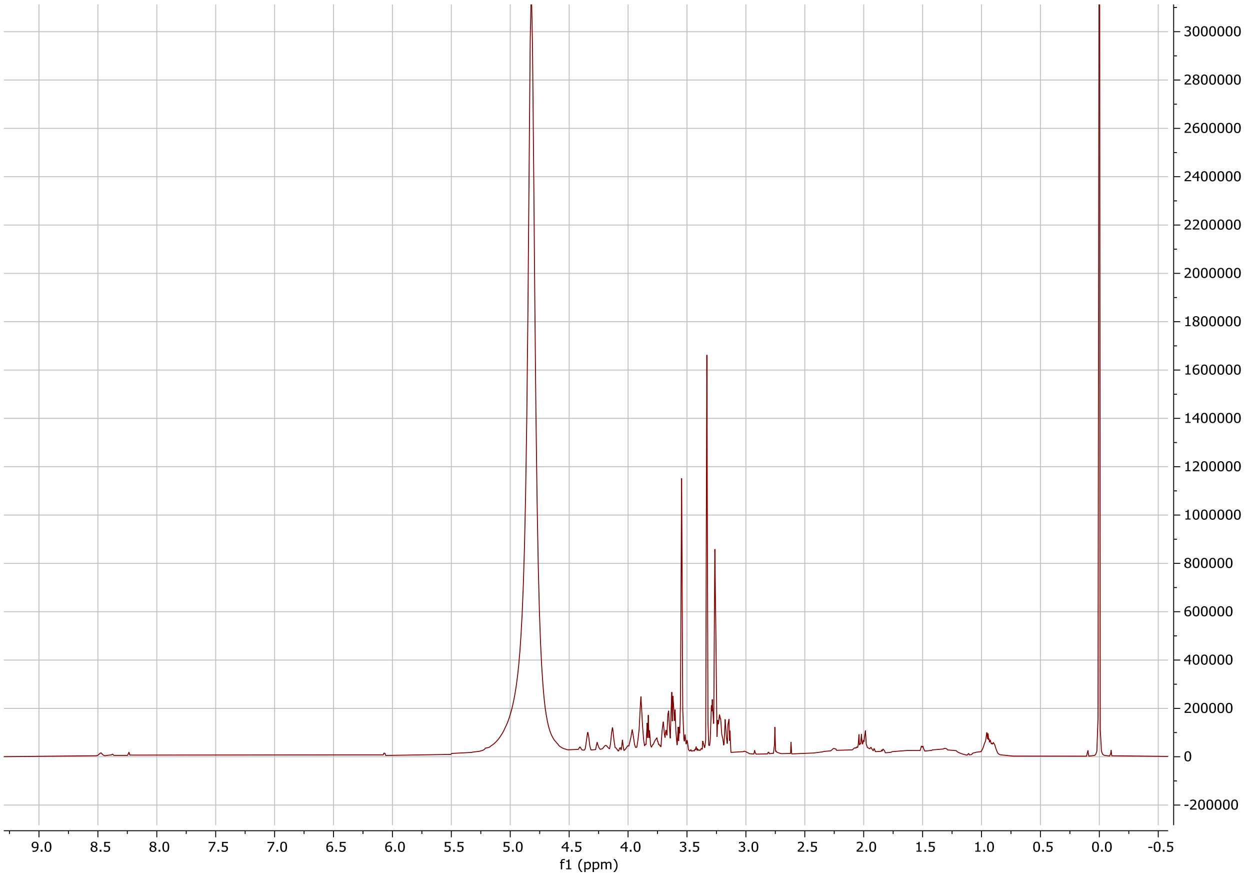

Figure S37: ^{1}H NMR spectrum for screening-guided isolation of fraction **PH-2**

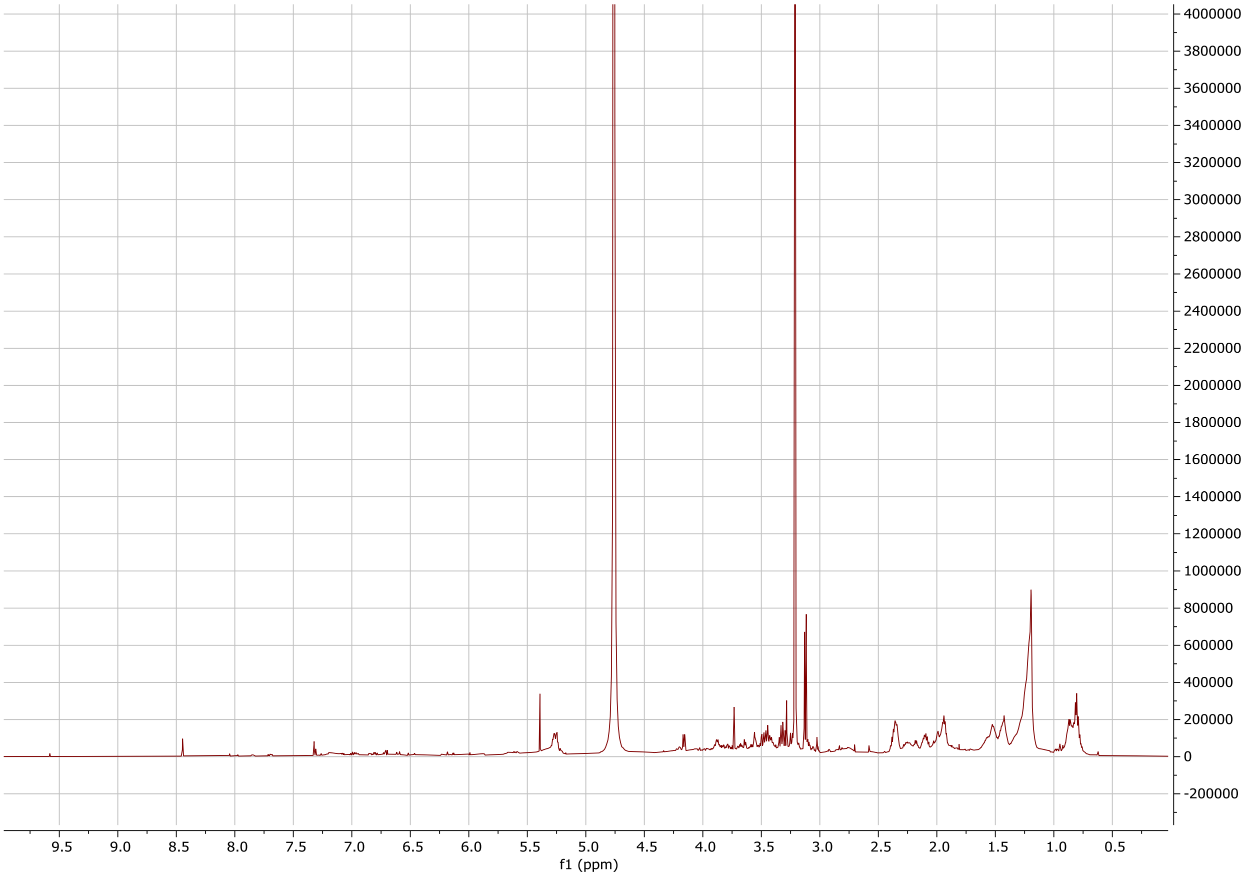

Figure S38: ^{1}H NMR spectrum for screening-guided isolation of fraction **PH-3**

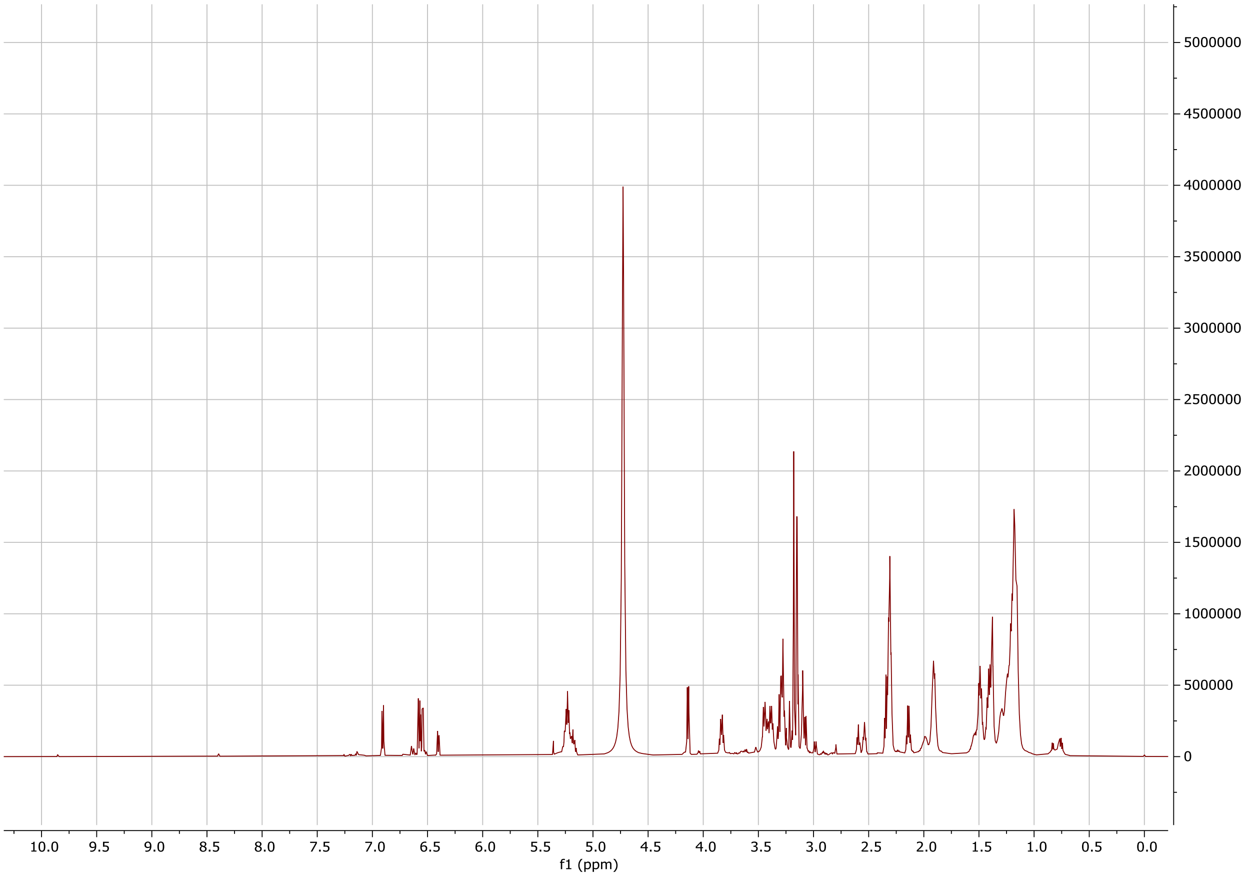

Figure S39: ^{1}H NMR spectrum for screening-guided isolation of fraction **PH-4**

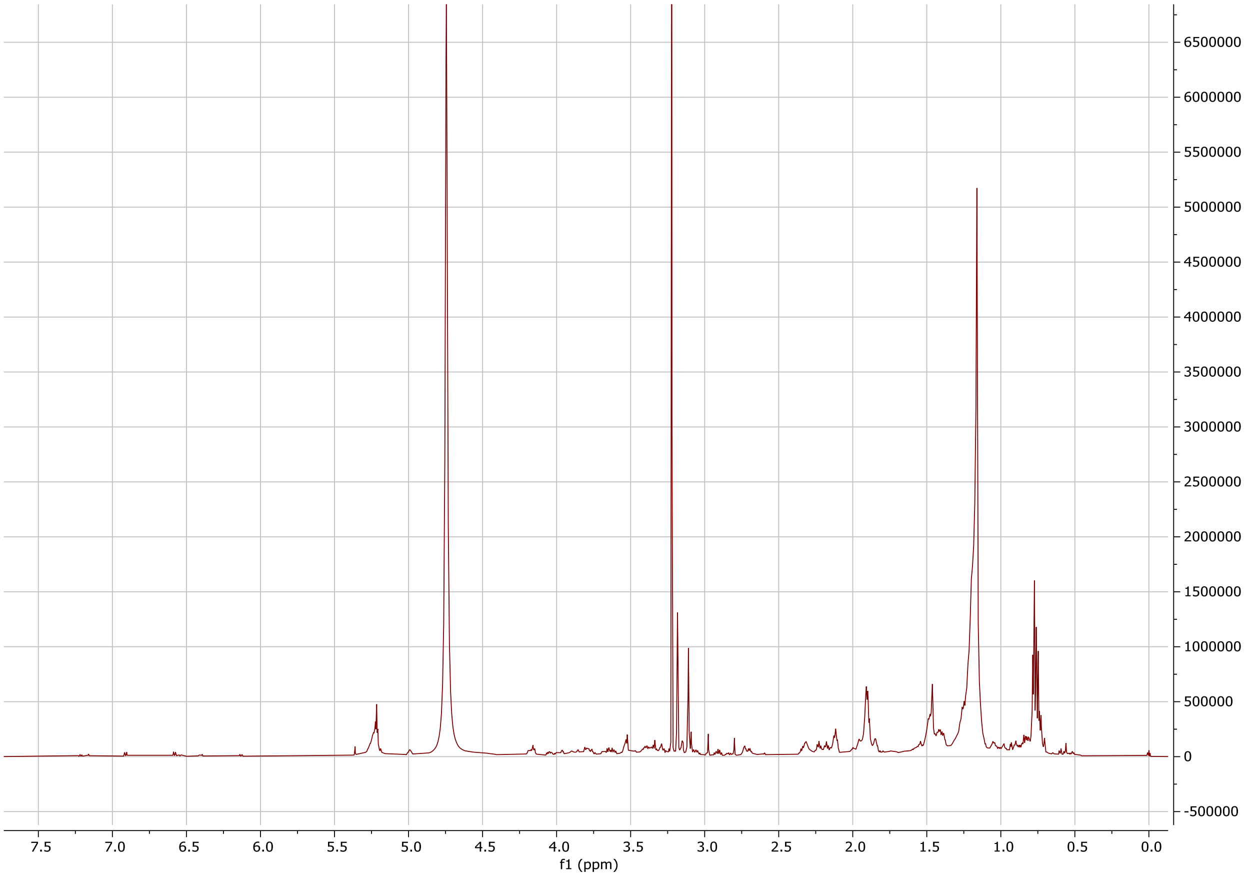

Figure S40: ^{1}H NMR spectrum for screening-guided isolation of fraction **PH-5**

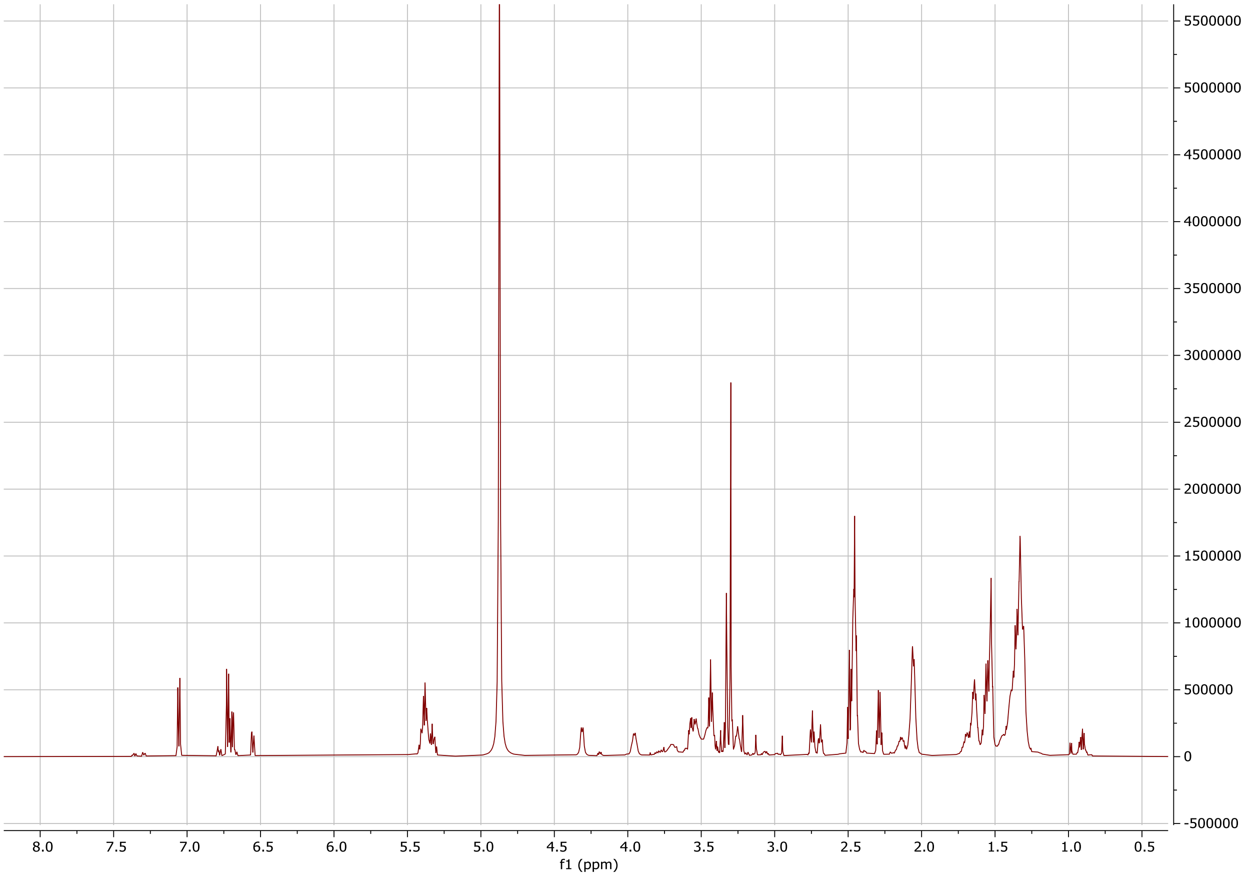

Figure S41: ^{1}H NMR spectrum for screening-guided isolation of fraction **PH-4-12**

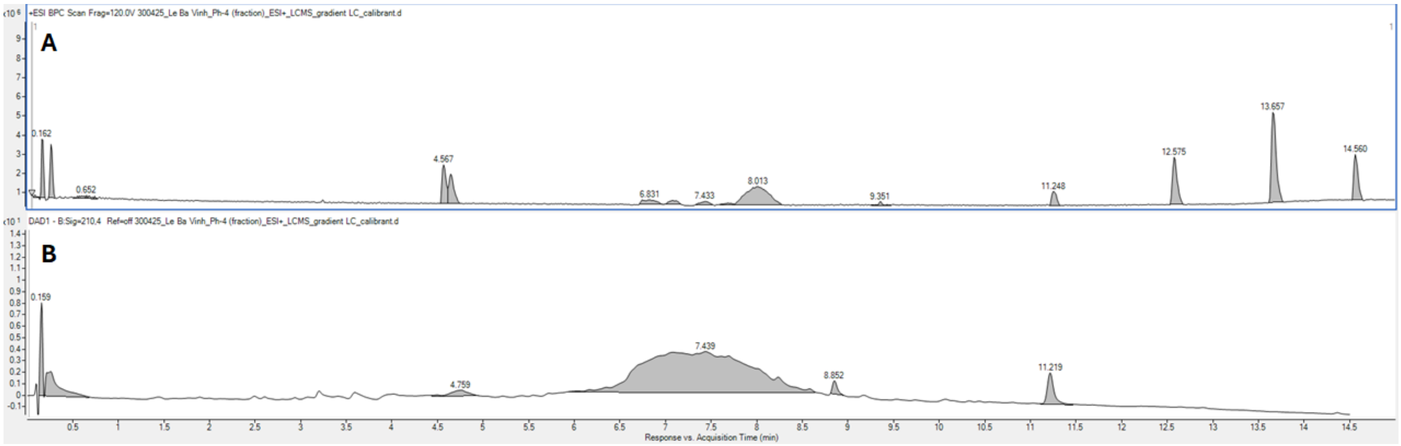

Figure S42: Secondary metabolites present in subfraction **PH-4-12** of the sponge *Phakellia* sp. were profiled using LC–QTOF–MS/MS in positive ionization mode. (A) Total ion chromatogram (TIC) acquired in positive mode. (B) Ultraviolet (UV, 220 nm) chromatogram of subfraction **PH-4-12**.
